# CHK1 inhibition exacerbates replication stress induced by IGF blockade

**DOI:** 10.1038/s41388-021-02080-1

**Published:** 2021-11-12

**Authors:** Xiaoning Wu, Elena Seraia, Stephanie B. Hatch, Xiao Wan, Daniel V. Ebner, Francesca Aroldi, Yanyan Jiang, Anderson J. Ryan, Thomas Bogenrieder, Ulrike Weyer-Czernilofsky, Guillaume Rieunier, Valentine M. Macaulay

**Affiliations:** 1 Department of Oncology, University of Oxford, Oxford, UK; 2 Target Discovery Institute, University of Oxford, Oxford, UK; 3 CRUK/MRC Oxford Institute for Radiation Oncology, University of Oxford, Oxford, UK; 4 Boehringer Ingelheim RCV GmbH & Co KG, Vienna, Austria; 5 AMAL Therapeutics, c/o Fondation pour Recherches Médicales, 1205 Geneva, Switzerland; 6 Department of Urology, University Hospital Grosshadern, Ludwig-Maximilians-University, Munich, Germany; 7 Nuffield Department of Surgical Sciences, University of Oxford, Oxford, UK; 8 Present Address: Immunocore Ltd, Abingdon, UK

**Keywords:** High-throughput screening, Targeted therapies

## Abstract

We recently reported that genetic or pharmacological inhibition of insulin-like growth factor receptor (IGF-1R) slows DNA replication and induces replication stress by downregulating the regulatory subunit RRM2 of ribonucleotide reductase, perturbing deoxynucleotide triphosphate (dNTP) supply. Aiming to exploit this effect in therapy we performed a compound screen in five breast cancer cell lines with IGF neutralising antibody xentuzumab. Inhibitor of checkpoint kinase CHK1 was identified as a top screen hit. Co-inhibition of IGF and CHK1 caused synergistic suppression of cell viability, cell survival and tumour growth in 2D cell culture, 3D spheroid cultures and in vivo. Investigating the mechanism of synthetic lethality, we reveal that CHK1 inhibition in IGF-1R depleted or inhibited cells further downregulated RRM2, reduced dNTP supply and profoundly delayed replication fork progression. These effects resulted in significant accumulation of unreplicated single-stranded DNA and increased cell death, indicative of replication catastrophe. Similar phenotypes were induced by IGF:WEE1 co-inhibition, also via exacerbation of RRM2 downregulation. Exogenous RRM2 expression rescued hallmarks of replication stress induced by co-inhibiting IGF with CHK1 or WEE1, identifying RRM2 as a critical target of the functional IGF:CHK1 and IGF:WEE1 interactions. These data identify novel therapeutic vulnerabilities and may inform future trials of IGF inhibitory drugs.

## Introduction

Many cancers show aberrant signalling via the insulin-like growth factor (IGF) axis, activating type 1 IGF receptors (IGF-1Rs) and variant insulin receptors (INSRs) to signal via phosphatidylinositol 3-kinase–AKT–mammalian target of rapamycin (PI3K-AKT-mTOR) and mitogen-activated protein kinase kinase–extracellular signal-regulated kinases (MEK-ERK) [1]. Through these effectors, IGFs mediate cell cycle progression, cancer cell proliferation and protection from apoptosis [1–3]. Previous studies from our group and others revealed that IGF-1R blockade sensitises human tumour cells to ionising radiation (IR) and cytotoxic drugs [4–9]. We further reported that IGF-1R depletion or inhibition delays repair of IR-induced DNA double-strand breaks (DSBs), and inhibits DSB repair via both homologous recombination (HR) and non-homologous end-joining [5, 6].

The present study was underpinned by three observations. First, given evidence that IGFs regulate the response to IR, we also found evidence that IGF-1R depletion induced endogenous DNA lesions marked by γH2AX foci in prostate cancer cells [10]. The origin of these lesions was unclear, although γH2AX foci are known to accumulate at DSBs and stalled replication forks to recruit repair and cell signalling machineries, serving as a sensitive indicator of DNA damage and replication stress [11, 12]. Secondly, we noted that an IGF gene signature identified in MCF7 breast cancer cells included components of the replication machinery [13]. Thirdly, we recently identified an absolute requirement for IGF-1 to maintain replication integrity by regulating the function of ribonucleotide reductase [14], the rate-limiting step for dNTP production [15]. RNR contains two subunits: ribonucleotide reductase subunit M1 (RRM1) and M2 (RRM2) [16]. Acting via both PI3K-AKT and MEK-ERK-JUN pathways, we showed that IGF-1 potently upregulates RRM2 transcription [14]. Thus, IGF-1R inhibited or depleted cells downregulate RRM2 and dNTP supply, delaying replication fork progression, activating ATR/CHK1 and the replication checkpoint, and suppressing new origin firing [14], all key hallmarks of replication stress [17]. The resulting single-stranded DNA (ssDNA) lesions were found to be marked by γH2AX foci and 53BP1 nuclear bodies, which form in G1 phase to protect from erosion under-replicated DNA generated by mitotic transmission of chromosomes under replication stress [18, 19]. Finally, we showed that ssDNA lesions are converted to toxic DSBs in cells lacking functional ataxia telangiectasia mutated (ATM), likely due to failure to form 53BP1 bodies and/or a role for ATM in SSB repair or fork protection [14].

While striking, the replication stress phenotype induced by IGF blockade appeared tolerable with minor impact on viability. Hypothesising that this state represents an exploitable vulnerability, we conducted a compound screen using IGF neutralising antibody xentuzumab, currently undergoing clinical evaluation with evidence of benefit in patients with oestrogen receptor positive (ER+) breast cancer and non-visceral metastases [20–22]. We tested five ER+ breast cancer cell lines with xentuzumab alone or with a compound library of inhibitors targeting cell cycle controls, replication and repair. Our recent report described screen outcomes in MCF7 cells [14]; here we describe the findings in the full cell line panel. We show that tolerable replication stress in IGF-inhibited cells is exacerbated by co-targeting IGF with CHK1 or WEE1 due to profound RRM2 protein depletion, consistent with roles for these checkpoint kinases in maintaining E2F1-mediated RRM2 transcription and counteracting CDK-mediated RRM2 degradation [23–25]. This approach represents a potential treatment strategy that induces intolerable replication stress, replication catastrophe and tumour cell death.

## Results

### IGF axis inhibition induces tolerable replication stress associated with therapeutic vulnerabilities

**Fig. 1:**
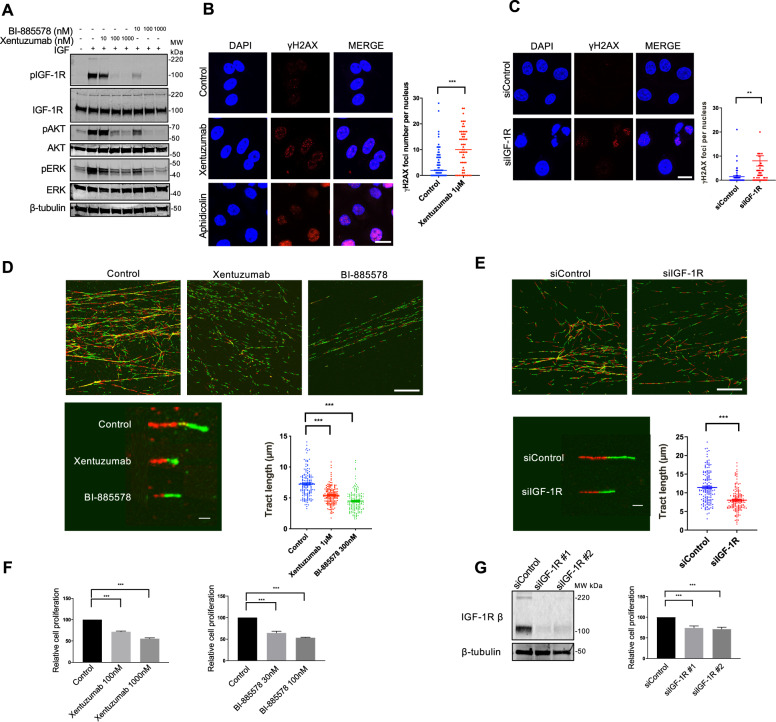
IGF blockade induces tolerable replication stress. **A** Western blot analysis of MCF7 cells exposed to xentuzumab or BI-885578 for 4 days, serum starved for 24 h in the presence of the same inhibitors and stimulated with 50 nM IGF-1 for 15 min. **B** Representative image of γH2AX immunostaining in MCF7 cells treated with 1 µM xentuzumab or 0.3 µM aphidicolin for 72 h. Scale bar: 20 μm. Graph to right: quantification of γH2AX (>50 cells). Data represent mean ± SEM, pooled from three independent experiments. **C** Representative image of γH2AX immunostaining in MCF7 cells transfected with siControl or siIGF-1R for 48 h. Scale bar: 20 μm. Graph to right: quantification of γH2AX (>50 cells per condition). **D** Representative images of DNA fibre tracts (CIdU, red; IdU, green) in MCF7 treated with xentuzumab (1 μM) or BI-885578 (300 nM) for 24 h. Scale bar: 20 μm. Graph to right: quantification of fibre tract length (>150 tracts) analysed using ImageJ software. **E** Representative images of DNA fibres in MCF7 cells transfected with siControl or siIGF-1R for 48 h. Scale bar: 20 μm. Quantification of fibre tract length (>150 tracts) shown on the right. **F** Cell viability tested 5 days after drug treatment (CellTiter Blue assay) expressed as % viability of solvent-treated controls. **G** Western blot analysis of MCF7 cells transfected with siControl or siIGF-1R and lysed after 24 h (left). Transfected cells were collected 24 h later and reseeded for cell viability assay (5 days after 24 h transfection). Results were expressed as % viability of siControl-transfected cells (right). *P < 0.05; **P < 0.01; ***P < 0.001.

Using genetic and pharmacological approaches to block IGF signalling, we recently uncovered a previously-unrecognised role for IGFs in regulating global DNA replication, with replication stress upon IGF axis blockade [14]. To confirm this effect, we first tested IGF ligand antibody xentuzumab (BI-836845), and IGF-1R tyrosine kinase inhibitor BI-885578 [20, 26] in MCF7 breast cancer cells. Both drugs caused dose-dependent inhibition of IGF-induced phosphorylation of IGF-1R, AKT and ERKs (Fig. 1A). We observed significant increase in γH2AX foci in xentuzumab-treated MCF7 cells (Fig. 1B). The foci were comparable in size and intensity to foci induced by aphidicolin that causes replication stress by inhibiting replicative DNA polymerases [27]; some aphidicolin-treated cells also exhibited pan-nuclear γH2AX, suggesting DNA damage-induced apoptosis [28]. Accumulating γH2AX foci were also induced by IGF-1R depletion in MCF7 cells (Fig. 1C), and by xentuzumab treatment in a second ER+ breast cancer cell line, ZR-75-1 (Supplementary Fig. S1A), consistent with our previous findings in prostate and breast cancer cells [10, 14]. To investigate this initial evidence of replication stress, we assessed replication fork dynamics. Labelling newly-replicated DNA with 5-chloro-2′deoxyuridine (CIdU) and 5-iodo-2′deoxyuridine (IdU), DNA fibre assays enable quantification of the rate of fork progression, fork stalling and origin firing [29]. Significant shortening of DNA tracts was detected in MCF7 cells treated with xentuzumab or BI-885578, and in IGF-1R-depleted cells compared to siControls (Fig. 1D, E). There was no evidence of significant fork stalling (only CIdU labelling), increased origin firing (only IdU labelling, ref. [29], or sister fork asymmetry (Supplementary Fig. S1B–D). The latter finding suggested that replication fork delay was due to global reduction in replication rather than localised DNA lesions [30]. When we tested consequences for cell viability, MCF7 cells retained 50–70% viability of controls after exposure to xentuzumab or BI-885578, or IGF-1R depletion (Fig. 1F, G). These data confirmed our previous finding of significant but tolerable replication stress in IGF-inhibited or IGF-1R-depleted cells [14].

**Fig. 2:**
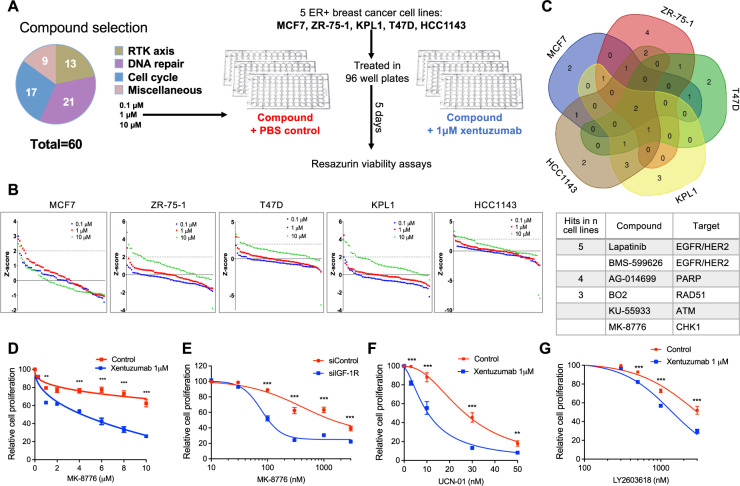
Compound screen identifies drug combination of CHK1 inhibitor and IGF inhibitor. **A** Compound library contained 59 small molecule drugs and controls (DMSO solvent for compounds, PBS for xentuzumab). MCF7, ZR-75-1, KPL1, T47D, HCC1143 were seeded in 96-well plates, treated with DMSO or library compounds at 0.1 µM, 1 µM, 10 µM with PBS or 1 µM xentuzumab and cell viability was determined after 5 days. **B** Cell viability data were used to calculate Z-scores as described in Supplementary Methods. Z-scores were ranked for all compounds for each cell line. Dotted line: Z-score = 2, threshold for hit identification. **C** Venn diagram showing overlap of hit compounds in five cell lines. Below: screen hits in at least three cell lines. **D** MCF7 cells were exposed to xentuzumab and MK-8776 for 5 days. **E** MCF7 cells were transfected with siControl or siIGF-1R for 24 h, and then exposed to solvent (control) or MK-8776 for 5 days. **F**, **G** MCF7 cells were exposed for 5 days to xentuzumab with UCN-01 (**F**) or LY2603618 (**G**) prior to cell viability assay. Data in (**D**–**G**) were expressed as % viability of solvent-treated control or siControl cells and represent mean ± SEM, pooled from *n* = 3 independent experiments. Two-way ANOVA of data in (**D**–**G**) indicated that both xentuzumab and IGF-1R depletion induced significant difference (*P* < 0.001) in the response to CHK1 inhibition; graphs show post-hoc analysis of significance at each drug concentration.

Aiming to enhance this phenotype to intolerable levels, we performed a compound screen to identify additive or synergistic drug combinations with xentuzumab. We chose to screen luminal ER+ breast cancer cell lines because xentuzumab is undergoing trials in ER+ breast cancer, with evidence of benefit in patients with non-visceral metastases [20–22]. MCF7, ZR-75-1, KPL1, T47D, and HCC1143 cell lines were tested against a custom compound library of inhibitors targeting cell signalling, cell cycle control, DNA replication and DNA damage responses (Fig. 2A, Supplementary Table S1). Compounds were tested at 0.1 µM, 1 µM and 10 µM in the absence or presence of 1 µM xentuzumab, which is near the steady-state circulating concentration at the dose selected for Phase II trials [21]. In initial Incucyte tests we optimised seeding densities and confirmed that xentuzumab caused detectable but relatively minor viability inhibition (Supplementary Fig. S2A). For the screens, cells were treated with compounds alone or with xentuzumab, or xentuzumab alone, and cell viability determined after 5 days (Fig. 2A). Calculated according to [31], screen Z-factor >0.5 indicates excellent screen quality, Z-factor 0–0.5 acceptable and Z-factor ≤0 inadequate (overlap between positive and negative controls). Using DMSO (solvent) as negative control and PLK inhibitor BI-2536 as positive control, most screens were excellent/acceptable, with Z-factors for MCF7 screens of ≥0.64, for T47D and HCC1143 ≥ 0.5, for ZR-75-1 0.24, 0.52, 0.65 at 0.1, 1 and 10 µM respectively. KPL1 screens gave Z-factors <0 at 0.1 and 1 µM and 0.48 at 10 µM, so only the latter was used to investigate hits. Compounds were ranked on Z-scores; those with Z-score >2 were identified as positive hits that sensitised to xentuzumab (Fig. 2B, Supplementary Table S2–6). Figure 2C shows the overlap of hits between cell lines, and below, the six top-ranked compounds. Screen hits in ≥3 cell lines included inhibitors of ATM but not ATR, confirming data from our recent report [14], and inhibitors of PARP, as reported by others [32]. Of the 6 compounds, the only agent not previously reported to be at least additive with IGF axis inhibition was CHK1 inhibitor MK-8776. This was also the only compound to achieve Z-score >3 with xentuzumab when comparing Z-scores of cell cycle/repair proteins CHK1, ATM, ATR and PARP. None of the compounds found to have Z-scores >2 with xentuzumab (Supplementary Table S7A) had Z-score >2 when tested alone in the same cell line (Supplementary Table S7B). MK-8776 was of particular interest having been shown to induce replication stress [23, 33, 34]. Therefore, we investigated the hypothesis that CHK1 has a protective role in the context of IGF inhibition.

MK-8776 was a screen hit in T47D, KPL1 and HCC1143 but not MCF7 or ZR-75-1 (Supplementary Table S2–6). However, these may have been false negatives: viability and clonogenic assays in MCF7 and ZR-75-1 showed evidence of a combination effect between MK-8776 and xentuzumab or IGF-1R depletion (Fig. 2D, E, Supplementary Fig. S2B, C). A similar combination effect was observed in KPL1 cells co-treated with MK-8776 and xentuzumab, confirming MK-8776 as a screen hit in this cell line, and HeLa cervical cancer cells (Supplementary Fig. S2D, E). MCF7 cells were also sensitised by BI-885578 to MK-8776 (Fig. S2F), and by xentuzumab to alternative CHK1 inhibitors LY2603618 and UCN-01 (Fig. 2F, G). These results using inhibitors of different classes provide support for functional interaction between CHK1 and IGF axis inhibition.

### CHK1 inhibition induces replication catastrophe in IGF-1R depleted cells

**Fig. 3:**
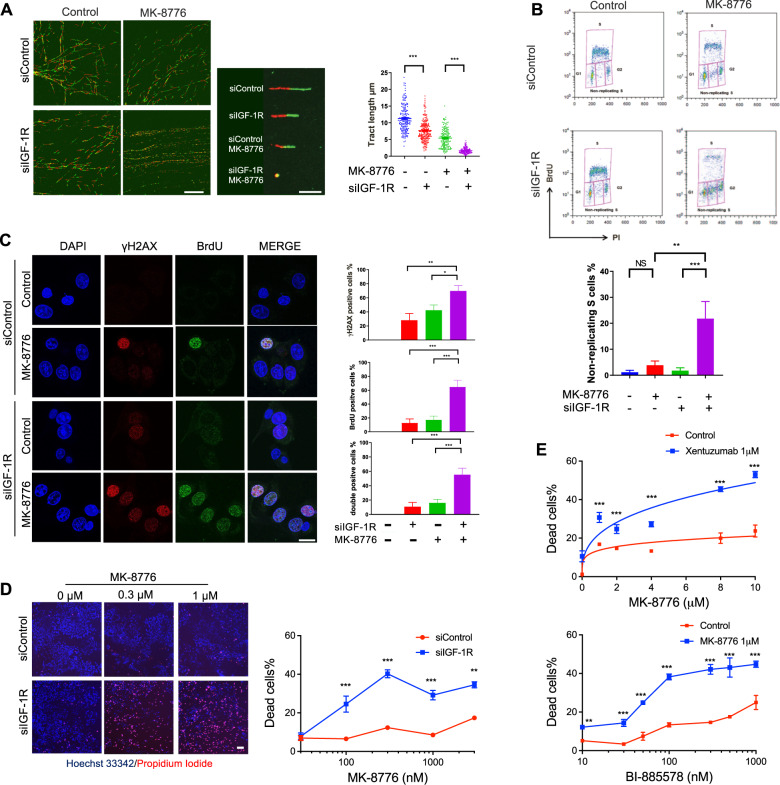
CHK1 inhibition induces replication catastrophe in IGF-1R depleted cells. **A** Representative images of DNA fibre tracts in MCF7 cells transfected with siControl or siIGF-1R for 24 h and then exposed to solvent or 300 nM MK-8776 for 24 h. Scale bar: 20 μm. Graph: quantification of fibre tract length (>150 tracts). **B** Flow cytometry analysis of cell cycle distribution of MCF7 cells after siRNA transfection for 24 h and then exposure to solvent or 300 nM MK-8776 for 24 h. Quantification of non-replicating S-phase cells is represented as mean ± SD, pooled from three independent experiments. These data were gated to exclude cells with >4 N DNA content; ungated data are shown in Supplementary Fig. S3E. **C** Representative images of BrdU and γH2AX immunofluorescence in MCF7 cells transfected with siControl or siIGF-1R for 24 h and then exposed to solvent or 300 nM MK-8776 for 24 h. Cells were cultured with 10 µM BrdU for 36 h before fixation and analysed in non-denaturing conditions to detect ssDNA. Scale bar: 20 µm. Graphs to right: upper, γH2AX-positive cells (>10 foci + pan-nuclear staining); centre, BrdU positive cells (>5 foci + pan-nuclear staining); lower, double-positive cells; ≥10 images were quantified. **D** Representative images of PI/Hoechst 33342 staining in MCF7 cells transfected with siControl or siIGF-1R for 24 h and then exposed to solvent or MK-8776 for 5 days. Scale bar: 200 μm. Graph below: dead cells % expressed as % PI-positive cells/Hoechst positive cells. Data represent mean ± SEM, pooled from three independent experiments. **E** MCF7 cells were exposed to xentuzumab (upper) or BI-885578 (lower) in combination with solvent or MK-8776 for 5 days. Dead cells expressed as mean ± SEM % PI-positive cells/Hoechst positive cells. For (**D**, **E**) there was a significant difference between each of the dose–response curves (*P* < 0.001) by 2-way ANOVA.

The ATR/CHK1 pathway plays a critical role in mediating replication stress responses during S phase [23, 35, 36]. We hypothesised that targeting CHK1 would exacerbate replication stress and viability inhibition induced by IGF blockade. In DNA fibre assays DNA fibre tract shortening was induced by MK-8776 or IGF-1R depletion, the latter effect consistent with Fig. 1E, and MK-8776 caused highly significant fibre shortening in IGF-1R depleted cells (Fig. 3A), indicating extreme replication fork delay. ATR-CHK1 inhibition is reported to induce unscheduled origin firing [37], and indeed, MK-8776 promoted origin firing in MCF7 cells (Supplementary Fig. S3A). Given the reported ability of ATM loss to sensitise to ATR/CHK1 inhibition [38], and our recent finding that ATM loss synergises with IGF axis inhibition [14], we also tested ATM-deficient SK-CO-1 colorectal cancer (CRC) cells [39]. Here, MK-8776 or IGF-1R depletion caused replication fork delay, while the combination induced dramatic suppression of fork progression without additive effect on new origin firing (Supplementary Fig. S3B). We also performed fibre assays in MCF7 cells after 24 h exposure to MK-8776, xentuzumab or BI-885578. Separately, each agent caused significant replication delay, and MK-8776 increased newly-fired origins, while addition of xentuzumab or BI-885578 to MK-8776 resulted in much shorter DNA fibres (Supplementary Fig. S3C, D).

These data suggested that IGF:CHK1 co-inhibition dramatically suppressed DNA replication. This effect was associated with significant increase in non-replicating S-phase cells, those with DNA content between 2N and 4N without incorporation of 5-Bromo-2′-deoxyuridine (BrdU), in IGF-1R depleted MK-8776-treated cells (Fig. 3B). To determine whether these treatments had induced polyploidy, which could cause the apparent increase in BrdU-negative cells with DNA content between 2N and 4N, we also checked ungated data. After IGF-1R depletion alone there were fewer than 1% polyploid cells, with no increase upon addition of MK-8776 (Supplementary Fig. S3E). This suggests that there was a genuine increase in non-replicating S-phase cells, and that S-phase transit was indeed severely compromised. We then performed double immunostaining assays for two replication stress consequences: γH2AX and ssDNA, the latter visualised by BrdU labelling and detection under non-denaturing conditions [23]. Focal and pan-nuclear γH2AX are established markers of replication stress-induced strand breaks and apoptosis [11, 28, 40]. Quantifying γH2AX-positive cells as those with >10 foci or pan-nuclear staining as [11], MK-8776 treatment of siControl transfectants enhanced focal and pan-nuclear γH2AX signal (Fig. 3C), consistent with previously reported results upon UCN-01 treatment [41], with significant increase upon IGF-1R depletion (Fig. 3C upper graph). Native BrdU staining (ssDNA) was also significantly increased by this combination (Fig. 3C middle). Quantifying cells double positive for γH2AX and BrdU revealed evidence of a greater than additive effect upon MK-8776 treatment of IGF-1R depleted cells (Fig. 3C, lower), consistent with high levels of replication stress and replication catastrophe [23, 35]. Investigating the consequences of this phenotype, we quantified cell death using Hoechst 33342, which stains both live and dead cells, and propidium iodide (PI) that binds DNA only in dead cells [42]. IGF-1R depletion or 1 µM xentuzumab induced death of <10% MCF7 cells, whereas 1 µM BI-885578 caused 27 ± 1.22% cell death (Fig. 3D, E), possibly reflecting more potent IGF-1R inhibition (Fig. 1A) and/or additional INSR inhibition [26]. Cell death was significantly increased by addition of MK-8776 in IGF-1R depleted cells and cells treated with xentuzumab or BI-885578 (Fig. 3D, E). Together, these data strongly suggest that CHK1 inhibition enhanced replication stress induced by IGF blockade to intolerable levels, triggering replication catastrophe and cell death.

### CHK1 inhibition exacerbates RRM2 downregulation and dNTP pool reduction induced by IGF-1R depletion, with rescue by RRM2 overexpression

**Fig. 4:**
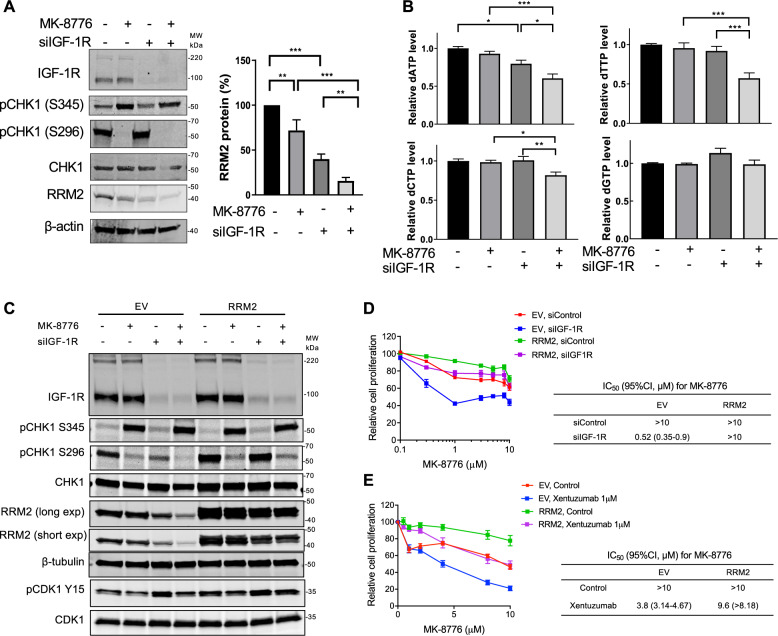
CHK1 inhibition reduces RRM2 protein levels and decreases dNTP pools in IGF-1R depleted cells. **A** Western blot analysis of MCF7 cells transfected with siControl or siIGF-1R for 24 h, and exposed to solvent or 300 nM MK-8776 for 24 h. Graph: RRM2 protein levels were quantified by ImageJ, corrected for β-tubulin loading and expressed as percent RRM2 protein content of solvent-treated controls. Data represent mean ± SEM from three independent experiments. **B** dNTPs extracted from cells in (**A**) were assayed, results were normalised to solvent controls (left bar) and represent mean ± SEM, pooled from three independent experiments. **C** Empty vector (EV) control cells and RRM2-overexpressing cells were transfected with siControl or siIGF-1R for 24 h and then exposed to solvent or 300 nM MK-8776 for 24 h, followed by protein extraction and western blot analysis. **D** EV controls and RRM2-overexpressing cells were siRNA transfected as (**C**) and then exposed to solvent or MK-8776 for 5 days, followed by cell viability assay. Results were expressed as % viability of solvent-treated control. IC_50_ values and 95% confidence intervals (CI) were calculated from dose–response curves. By 2-way ANOVA there were significant differences in response to MK-8776 in siControl vs. siIGF-1R transfected EV cells, siControl vs. siIGF-1R transfected RRM2-overexpressing cells and siControl EV cells vs. siControl RRM2 transfectants (*P* < 0.001 for each comparison) but no significant difference in siControl EV cells vs. siIGF-1R transfected RRM2-overexpressing cells. **E** EV control cells and RRM2-overexpressing cells were exposed to solvent or MK-8776 in the presence or absence of 1 µM xentuzumab for 5 days followed by assay of cell viability, expressed as % of solvent-treated controls, showing IC_50_ values and 95% CI. Two-way ANOVA showed significant differences in response to MK-8776 in solvent controls vs. xentuzumab-treated EV cells, controls vs. xentuzumab-treated RRM2-overexpressing cells and control-treated EV vs. RRM2-overexpressing cells (*P* < 0.001 for each comparison) and no significant difference in the response of control-treated EV cells vs. xentuzumab-treated RRM2-overexpressing cells.

Given our finding that IGF-1R blockade causes transcriptional RRM2 downregulation [14], and reports that ATR/CHK1 inhibition downregulates RRM2 protein [23, 24], we assessed effects of IGF:CHK1 co-targeting on RRM2. Treating MCF7 cells with MK-8776 after siRNA transfection, we found that MK-8776 suppressed CHK1 autophosphorylation on S296, which targets CDC25 phosphatases to arrest cell cycle progression [43], verifying MK-8776 bioactivity, and increased S345-CHK1 phosphorylation (Fig. 4A). The latter effect is likely a feedback consequence of CHK1 inhibition, which deregulates CDK activity leading to increased origin firing, nucleotide depletion and fork breakage, triggering ATR activation [41]. MK-8776 or IGF-1R depletion caused moderate RRM2 protein reduction, with further reduction when these treatments were combined (Fig. 4A). RRM2 is a key component of the RNR complex, required to convert NDPs into dNDPs [16]. Therefore, we next investigated effects on dNTP content, having previously found in HLPC-based assay of MCF7 cell extracts that IGF-1R depletion reduced dATP with no change in dTTP or dGTP, and dCTP was undetectable [14]. Here, we modified an alternative assay [44] based on incorporation of tritium-labelled dNTPs into template DNA, using commercially-sourced dNTPs as controls (Supplementary Fig. S4A–D). All dNTPs were detectable in MCF7 extracts, and consistent with our previous results [14] IGF-1R depletion reduced only dATP (Fig. 4B). Combining MK-8776 and IGF-1R depletion further suppressed dNTP availability with significant reduction in dATP, dTTP and dCTP (Fig. 4B), although the changes were relatively modest.

To assess the association of these phenotypes with RRM2 downregulation we generated MCF7 cells stably expressing empty vector (EV) or RRM2. IGF-1R depletion reduced RRM2 protein in EV controls (Fig. 4C) as in parental MCF7 (Fig. 4A), and also in RRM2-overexpressing cells likely due to downregulation of endogenous RRM2, although residual RRM2 still exceeded levels in siControl-transfected EV controls (Fig. 4C). As before (Fig. 4A), MK-8776 blocked phospho-S296 CHK1 and increased phospho-S345 CHK1. In EV controls RRM2 was profoundly reduced by combining MK-8776 with IGF-1R depletion, comparable to the effect in parental MCF7 cells (Fig. 4A), while RRM2-overexpressing cells maintained RRM2 levels after CHK1 inhibition and IGF-1R depletion (Fig. 4C). Testing consequences for cell viability, IGF-1R depletion shifted the MK-8776 dose–response curve to the left in EV controls (Fig. 4D), again consistent with results in parental cells (Fig. 2C), while RRM2 overexpression shifted the curve to the right, with MK-8776 GI_50_ values >10 µM (Fig. 4D). IGF-1R depletion profoundly sensitised EV cells to MK-8776 but had no effect in RRM2-overexpressing cells, with GI_50_ remaining >10 µM (Fig. 4D). Similar results were observed on testing xentuzumab with MK-8776: in EV controls, xentuzumab reduced the MK-8776 GI_50_ from >10 µM to 3.8 µM, with a much smaller change (GI_50_ reduced from >10 to 9.6 µM) upon RRM2 overexpression (Fig. 4E). These data indicate almost complete rescue from suppression of cell viability upon IGF:CHK1 co-inhibition.

**Fig. 5:**
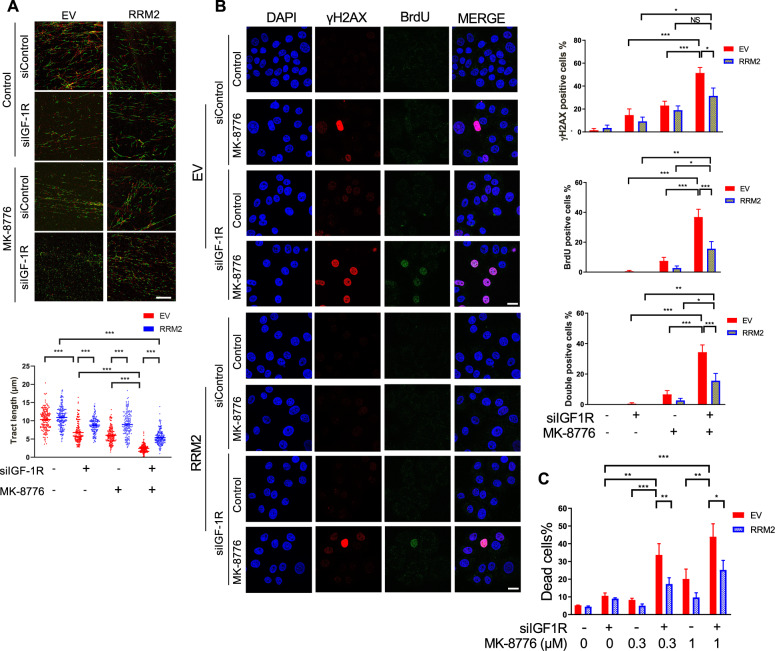
RRM2 overexpression rescues replication catastrophe induced by CHK1 inhibition and IGF-1R depletion. **A** Representative images of DNA fibre tracts in EV controls and RRM2-overexpressing cells transfected with siControl or siIGF-1R for 24 h and exposed to solvent or 1 µM MK-8776 for 24 h. Scale bar: 20 µm. Graph below: quantification of fibre tract length (>150 tracts). **B** Representative images of BrdU and γH2AX immunostaining in EV control cells and RRM2-overexpressing cells siRNA transfected and treated as (**A**), with addition of 10 µM BrdU for 36 h before fixation and analysis in non-denaturing conditions. Scale bar: 20 μm. Graphs to right: quantification of γH2AX-positive, BrdU positive and double-positive cells as in Fig. 3C; data represent mean ± SEM, pooled from 3 independent experiments. **C** EV controls and RRM2-overexpressing cells were transfected with siControl or siIGF-1R for 24 h and then exposed to solvent control or MK-8776 for 5 days, followed by PI/Hoechst 33342 staining to quantify dead cells, expressed as % PI-positive/Hoechst positive cells. Data represent mean ± SEM, pooled from three independent experiments each with three technical replicates.

We next investigated whether RRM2 overexpression influenced replication fork progression. In EV controls, MK-8776 or IGF-1R depletion alone caused moderate fibre shortening, while the combination caused extreme shortening (Fig. 5A) consistent with effects in parental MCF7 (Fig. 3A). In RRM2-overexpressing cells, DNA fibre tracts were significantly longer than EV controls upon IGF-1R depletion and MK-8776 treatment alone or in combination (Fig. 5A). This suggests that RRM2 overexpression rescued replication fork progression, although the rescue effect was partial: in RRM2-overexpressing cells mean fibre length upon combination treat was still shorter than in solvent-treated controls (Fig. 5A). RRM2 overexpression did not affect MK-8776-induced aberrant origin firing (Supplementary Fig. S5A) but rescued completely from accumulation of non-replicating S-phase cells in MK-8776-treated, IGF-1R depleted cells (Supplementary Fig. S4B). We also observed significant rescue from replication catastrophe, with fewer cells double-positive for γH2AX and ssDNA following MK-8776 treatment and IGF-1R depletion (Fig. 5B). This co-treatment induced significantly less cell death in RRM2-overexpressing cells compared with EV controls (Fig. 5C). Thus, RRM2 overexpression restored RRM2 protein, alleviated replication stress and rescued from cell death, suggesting that RRM2 is an important target of the interaction between CHK1 inhibition and IGF blockade.

### IGF-inhibited cells are sensitive to CHK1 inhibition in anchorage-independent growth

**Fig. 6:**
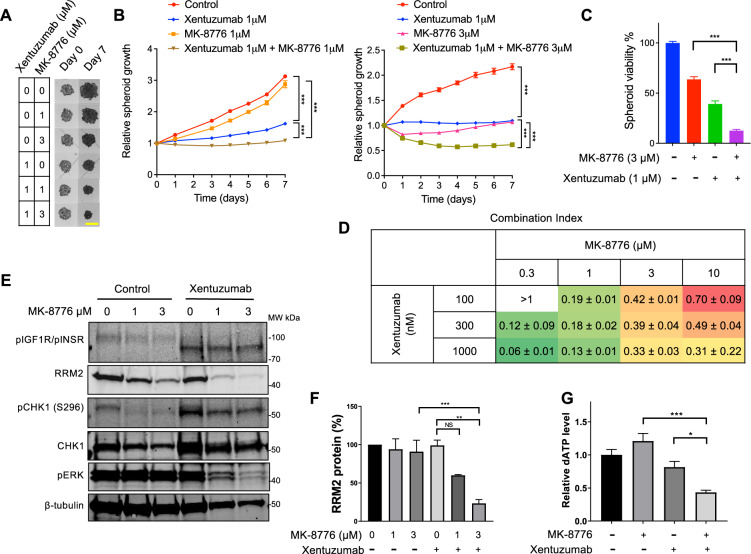
IGF-inhibited cells are sensitive to CHK1 inhibition in 3D spheroid models. **A** Representative images of SK-CO-1 spheroids exposed to xentuzumab alone or with MK-8776 for 7 days. Scale bar 2 mm. **B** Spheroid growth curves shown as fraction of Day 0 (pre-treatment) value. Two-way ANOVA showed a significant overall treatment effect and significantly reduced growth rates between xentuzumab or 1 or 3 µM MK-8776 alone and in combination (*P* < 0.001 for each comparison). **C** After 7 days, on completion of treatments and measurements in A-B, CellTiter Glo 3D viability assays were performed, and results expressed as mean ± SEM % solvent-treated controls pooled from *n* = 3 independent experiments. **D** CellTiter Glo 3D viability data were imported into CalcuSyn Software for CI value calculation. Data represent mean ± SD, pooled from three independent experiments. **E** Western blot analysis of SK-CO-1 spheroids (six spheroids per condition) exposed to solvent or MK-8776 in the presence or absence of 1 µM xentuzumab for 7 days. **F** RRM2 protein levels were quantified and adjusted for β-tubulin loading. Graph shows mean ± SEM RRM2 protein from three independent experiments, expressed as % of solvent-treated controls. **G** SK-CO-1 spheroids were treated with 1 µM xentuzumab and/or 3 µM MK-8776. Spheroid extracts were assayed for dATP and the results were normalised to solvent controls (left bar). Data represent mean ± SEM, pooled from three independent experiments.

Cancer cell spheroids recapitulate features of tumour growth in vivo, including increased cell-cell interactions and normoxic, hypoxic and necrotic zones reflecting tumour complexity [45–47]. MCF7 spheroids grew very slowly (~1.5-fold increase over 6 days) with no suppression by xentuzumab, partial inhibition by MK-8776, and a combination effect with xentuzumab at only 3 µM MK-8776 (Supplementary Fig. S6A). We examined alternative models as a preliminary to in vivo testing, and found that SK-CO-1 spheroids were sensitive to xentuzumab, consistent with our previous in vivo data [14], and were also inhibited by 3 µM MK-8776 alone (Fig. 6A, B). There was further growth inhibition in the presence of xentuzumab plus 3 µM MK-8776, with significant spheroid size regression and reduction in spheroid viability (Fig. 6B, C). HeLa spheroids were growth delayed by 1 µM xentuzumab or 3 or 10 µM MK-8776, showed almost complete growth arrest with the xentuzumab/MK-8776 combination, and a similar combination effect in response to BI-885578 plus MK-8776 (Supplementary Fig. S6B, C). We then tested another highly selective CHK1 inhibitor, SRA737 [48], which dose-dependently suppressed SK-CO-1 spheroid growth, with further significant suppression with 1 µM xentuzumab, including regression in the presence of 10 µM SRA737 and xentuzumab (Supplementary Fig. S6D). SRA737 also reduced cell viability in SK-CO-1 spheroids, with further reduction upon addition of 1 µM xentuzumab (Supplementary Fig. S5E). HeLa spheroid viability was largely unaffected by 1 µM xentuzumab or MK-8776 alone but showed a combination effect upon co-treatment of xentuzumab with 3 or 10 µM MK-8776 or 3 µM SRA737 (Supplementary Fig. S5F, G). Having previously reported that IGF targeting affects DNA damage responses in prostate cancer cells [6, 10], we also tested xentuzumab and MK-8776 in 22Rv1 prostate cancer spheroids, again finding a combination effect of this co-treatment on spheroid growth and viability (Supplementary Fig. S6H).

Given the sensitivity of SK-CO-1 spheroids to IGF:CHK1 co-inhibition (Fig. 6A, B), we tested xentuzumab and MK-8776 in mice bearing SK-CO-1 xenografts. In vivo, MK-8776 was administered in (2-Hydroxypropyl)-beta-cyclodextrin, as had been used in previous in vivo assessment of this drug [49, 50]. However, on starting treatment, tumour growth slowed even in controls treated with (2-Hydroxypropyl)-beta-cyclodextrin, which was previously reported to inhibit growth and enhance apoptosis [51]. Following 18 days treatment, combination-treated tumours were significantly smaller than the control and MK-8776 groups but not the xentuzumab alone group (Supplementary Fig. S6I, left). Off treatment, we observed regrowth of tumours in all groups except the combination treatment group, where mean tumour size was 23 ± 4% of controls, but the differences were not significant (Supplementary Fig. S6I, right). As an alternative route to assess combination effects we calculated Combination Indices (CI) from SK-CO-1 spheroid viability data using the Chou-Talalay method [52]. All combinations except the lowest concentrations (100 nM xentuzumab, 0.3 μM MK-8776) yielded CI values <0.8, with some values 0.1–0.3 indicating strong synergy between co-inhibition of IGF and CHK1 (Fig. 6D). We also tested for combination effects using Bliss Independence [53], expressing the results as predicted/observed fraction affected. All but one of the combinations generated values of <0.8, supporting a synergistic relationship between xentuzumab and MK-8776 (Supplementary Table S8).

To probe the mechanism of synergy we assessed RRM2 and dNTP levels in SK-CO-1 spheroids following 7 days treatment with xentuzumab and MK-8776 (Fig. 6A). In western blots of spheroid extracts, xentuzumab abolished ~98 kDa phospho-IGF-1R signal but induced 90–95 kDa signal, possibly phospho-INSR. RRM2 was largely unaffected by xentuzumab or MK-8776 alone but was significantly downregulated by combination treatment (Fig. 6E, F). In dNTP assays, given very small amounts of material, only dATP was detectable in SK-CO-1 spheroids, and was markedly reduced by the MK-8776 plus xentuzumab combination (Fig. 6G). It would have been advantageous to confirm these results in HeLa spheroids given the effect of this drug combination on spheroid growth (Supplementary Fig. S6B), but their smaller size prevented us from assaying dNTPs or other markers. None-the-less, the results in SK-CO-1 spheroids support our hypothesis that IGF:CHK1 co-targeting compromises dNTP supply.

### WEE1 inhibition induces replication catastrophe in IGF-1R depleted cells via RRM2 downregulation

**Fig. 7:**
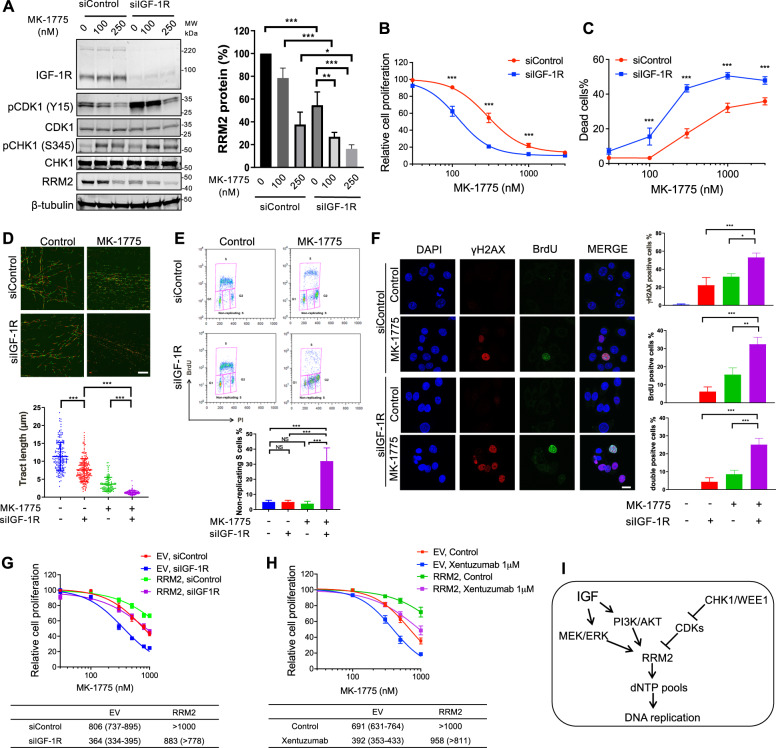
WEE1 inhibition induces replication catastrophe in IGF-1R depleted cells. **A** Western blot of MCF7 cells transfected with siControl or siIGF-1R for 24 h and exposed to solvent or MK-1775 for 24 h. Graph: RRM2 protein levels were quantified by ImageJ, corrected for β-tubulin and shown as mean ± SEM % of solvent-treated controls from 3 independent western blots. **B**, **C** MCF7 cells were transfected with siControl or siIGF-1R for 24 h and treated with solvent or MK-1775 for 5 days, followed by cell viability assay (**B**) or cell death assay (**C**), with significant differences (*P* < 0.001) in response to MK-1775 in both assays by 2-way ANOVA. **D** Representative images of DNA fibre tracts in MCF7 cells transfected with siControl or siIGF-1R for 24 h and exposed to solvent or 300 nM MK-1775 for 24 h. Scale bar: 20 μm. Graph below: quantification of fibre tract length (mean ± SEM of >150 tracts). **E** Analysis of MCF7 cell cycle distribution after transfection with siControl or siIGF-1R for 24 h and exposed to solvent or 300 nM MK-1775 for 24 h. Graph below shows quantification of non-replicating S-phase cells, mean ± SD, pooled from three independent experiments. **F** Representative images of native BrdU and γH2AX immunostaining in MCF7 cells transfected and treated as (**E**), and cultured with 10 µM BrdU for 36 h pre-fixation. Scale bar: 20 µm. Graphs to right: quantification of γH2AX positive, BrdU positive and double-positive cells as Fig. 3C; data represent mean ± SEM, pooled from three independent experiments. **G** EV controls and RRM2-overexpressing cells were transfected with siControl or siIGF-1R for 24 h, exposed to solvent or MK-1775 for 5 days, and cell viability was assayed. Data represent mean ± SEM, pooled from *n* = 3 independent experiments; below: IC_50_ values and 95% CI calculated from drug response curves. Two-way ANOVA showed similar differences in response to MK-1775 as in response to MK-8776 (Fig. 4D, E), with significant differences in siControl vs. siIGF-1R transfected EV cells, siControl vs. siIGF-1R transfected RRM2-overexpressing cells and siControl EV cells vs. siControl RRM2 transfectants (*P* < 0.001 for each comparison) but not in siControl EV cells vs. siIGF-1R transfected RRM2-overexpressing cells. **H** EV controls and RRM2-overexpressing cells were exposed to solvent or MK-1775 in the presence or absence of 1 µM xentuzumab for 5 days, followed by cell viability assay. Data represent mean ± SEM, pooled from *n* = 3 independent experiments with below, GI_50_ values and 95% CI. There were significant differences in response to MK-1775 in solvent controls vs. xentuzumab-treated EV cells, controls vs. xentuzumab-treated RRM2-overexpressing cells and control-treated EV vs. RRM2-overexpressing cells (*P* < 0.001 in each case) but not in control-treated EV cells vs. xentuzumab-treated RRM2-overexpressing cells. **I** RRM2 is regulated by IGFs and cell cycle checkpoint kinases CHK1 and WEE1, explaining profound RRM2 downregulation and replication catastrophe upon IGF:CHK1 or IGF:WEE1 co-inhibition.

Given our finding that IGF:CHK1 co-inhibition downregulates RRM2 inducing severe replication stress, we considered whether a similar response to IGF blockade is achieved by other compounds that influence RRM2. In our screen, the inhibitor of cell cycle checkpoint kinase WEE1, MK-1775, sensitised to xentuzumab only in KPL1 cells (Supplementary Table S2–6). In MCF7 cells, MK-1775 blocked inhibitory Tyr-15 CDK1 phosphorylation and reduced RRM2 protein, consistent with [25], and induced ATR-mediated phospho-S345-CHK1 phosphorylation (Fig. 7A), suggesting replication stress. IGF depletion increased phospho-Tyr-15 CDK1, not reported previously, suggesting WEE1 activation after IGF blockade. Notably, IGF-1R depletion plus MK-1775 resulted in further RRM2 downregulation (Fig. 7A), suppressed cell viability and induced excess cell death compared to siControls (Fig. 7B, C). Xentuzumab or BI-885578 also significantly sensitised MCF7 cells to MK-1775 in viability and clonogenic assays (Supplementary Fig. S7A–C). This suggests that MK-1775 was a false negative in the MCF7 screen, although the effect on response to MK-1775 was less marked than the sensitisation induced by xentuzumab to MK-8776 (Fig. 2D, Supplementary Fig. S2B). MK-1775 also markedly suppressed SK-CO-1 spheroid growth (Supplementary Fig. S7D), supporting a combination effect of co-inhibiting IGF and WEE1.

Consistent with effects on RRM2 protein, the combination of MK-1775 and IGF-1R depletion caused major replication tract delay, accumulation of cells in non-replicating S-phase and cells double-positive for γH2AX and ssDNA (Fig. 7D–F). Collectively, these results suggest that WEE1 inhibition exacerbated replication stress caused by IGF blockade, inducing replication catastrophe. To confirm the role of RRM2 downregulation in inducing these phenotypes, it would be advantageous to test for rescue by RRM2 overexpression. However, we did test effects of RRM2 overexpression on viability: comparable to data generated using MK-8776 (Fig. 4D, E), RRM2 overexpression rescued almost completely from the inhibitory effect on cell viability of the combination of MK-1775 with IGF-1R depletion or xentuzumab (Fig. 7G, H).

## Discussion

Here, we follow up our recent identification of a role for IGFs in regulating DNA replication [14] with approaches to exploit this effect in therapy. We confirm that IGF-1R depletion or IGF inhibition delayed replication fork progression, with accumulation of ssDNA lesions, non-replicating S-phase cells and ATR-CHK1 activation. This was a striking replication stress phenotype, but the effect was largely tolerable. In our previous report, we identified ATM loss as synergistic with IGF inhibition due to conversion of tolerable ssDNA lesions into toxic DSBs [14]. The current data identify a different mechanism of synergy, which we show is due to exacerbation of RRM2 downregulation by co-inhibition of CHK1 or WEE1, the IGF-CHK1 inhibitor combination being more effective. We previously reported that response to IGF-1R inhibition was enhanced by suppressing HR in prostate cancer models [10]. Here, we find no evidence that sensitivity to IGF:CHK1 co-inhibition associates with HR status, given our detection of combination effects in HR proficient MCF7, T47D, ZR-75-1, HCC1143 and HeLa cells [54–56], and SK-CO-1 and 22Rv1 that are HR deficient judging by PARP inhibitor sensitivity [39, 57]. Testing cell lines of different genotypes including immortalised, non-transformed cells, would be necessary to confirm this. CHK1 regulates RRM2 at the transcriptional level via E2F1, and both CHK1 and WEE1 inactivate CDKs, preventing RRM2 being targeted for proteasomal degradation, thus playing an important role in maintaining dNTP pools [23–25, 58].

Given these roles for ATM and CHK1, and evidence of ATR-CHK1 activation in replication-stressed cells, we had predicted that ATR inhibition would also synergise with IGF blockade. Others have reported additive-to-synergistic relationships between ATR inhibition and IGF-1R inhibitors BMS-754807 (CI values ≥0.777) and OSI-906 (CI ≥ 0.818) in MCF7 cells, with a stronger effect (CI ≥ 0.50) in cells induced to be BMS-754807 resistant [59]. We previously found no additivity/synergy between xentuzumab and ATR inhibition in MCF7 [14], and confirm here that ATR inhibition was not a hit in any of the 5 screened breast cancer cell lines, while in contrast we find evidence of synergy with CHK1 inhibition. This apparent paradox could relate to data showing or implying that ATR and CHK1 may not always function in tandem, including a report of dissociated ATR:CHK1 function in the context of UV-induced replication stress, synthetic lethality between CHK1 inhibition and ATR inhibition, also suggesting differential function, and identification of an apparently ATR-independent function for CHK1 related to bypass of replication barriers [60–62]. Of relevance here, ATR inhibition is reported to induce moderate ssDNA in most S-phase cells. This triggers a DNA-PK/CHK1-mediated backup pathway to suppress origin firing, creating a threshold such that ATR inhibition selectively kills cells under high replication stress, and CHK1 inhibition at a lower threshold [23].

We confirmed the central role of RRM2 in the synthetic lethality of IGF:CHK1 and IGF:WEE1 co-inhibition in an RRM2 overexpression model. Expression of constitutive RRM2 rescued from the major hallmarks of replication stress, including slowing of the replication fork, accumulation of ssDNA and non-replicating S-phase cells, and also prevented replication catastrophe. These data support the hypothesis that IGF-1, like CHK1 and WEE1, plays a key role in RRM2 regulation (Fig. 7I). IGF axis inhibitor trials have been compromised by lack of predictive biomarkers, although many trials included patients who obtained benefit, prompting intense efforts to characterise sensitive tumours and design rational drug combinations. Our data reveal CHK1 as a potential partner for co-inhibition with IGF blockade; we suggest that the efficacy of this combination may be most appropriately tested in ATM null tumours given our recent identification of ATM loss as a candidate biomarker for sensitivity to IGF axis blockade [14]. CHK1 inhibitors including SRA737 are being tested clinically [63, 64], suggesting that there may be merit in evaluating these agents with IGF axis blockade.

In summary, we highlight the critical role of IGF signalling in mediating DNA replication by regulating RRM2 and dNTP supply, and show that IGF-inhibited cells exhibit tolerable replication stress that represents a therapeutic vulnerability. We identify approaches to exploit this effect by co-inhibiting checkpoint kinases CHK1 or WEE1 to induce cancer cell death through replication catastrophe.

## Materials and methods

### Cell lines and reagents

MCF7, ZR-75-1, T47D, and HCC1143 breast cancer cell lines were from Dr Anthony Kong, King’s College London. RRM2-overexpressing MCF7 cells were generated as described [14]. HeLa cells were from Professor Adrian Harris, University of Oxford, 22Rv1 from Professor Sir Walter Bodmer, University of Oxford, KPL1 cells were purchased from European Collection of Authenticated Cell Cultures and SK-CO-1 from American Type Culture Collection. Cell lines were negative for mycoplasma infection (MycoAlert, Lonza) and were authenticated by STR genotyping at Cancer Research UK Clare Hall Laboratories and Eurofins. Early passage stocks were expanded and cryopreserved and used within 20 passages of recovery. Xentuzumab and BI-885578 were obtained from Boehringer Ingelheim. MK-8776, SRA737 and LY2603618 were purchased from Selleck Chemicals, UCN-01 from Cambridge Bioscience, MK-1775 from Axon Medchem and aphidicolin from Sigma-Aldrich.

*Viability, death and clonogenic assays* were performed as described [6, 14]. Viability data were analysed in Graphpad Prism 8 to calculate half-maximal inhibitory concentrations (IC_50_).

*Compound screens* were performed in duplicate using a 60-compound custom library (Supplementary Table S1) at 0.1 µM, 1 µM, 10 µM alone or with PBS (control) or 1 µM xentuzumab for 5 days. Cell seeding, treatment and viability assay were as [14]. Z-factors (screen quality) and Z-scores (compound ranking) were calculated as described [31, 65] and Supplementary Methods.

*Gene silencing, western blot, flow cytometry, DNA fibre assays* were performed as [14] using AllStars Negative Control siRNA, IGF-1R siRNA #1 (S100017521) and #2 designed in-house [66], all from Qiagen, and antibodies listed in Supplementary Table S9.

*Immunofluorescence for γH2AX and ssDNA:* as described in [23], ssDNA was detected by BrdU staining under non-denaturing conditions. Cells were pulsed with 10 µM BrdU (Sigma-Aldrich) and 36 h later fixed with 4% paraformaldehyde for 12 min, permeabilized using TFT buffer (0.1% Triton X-100, 4% FBS in phosphate-buffered saline, PBS) for 5 min and blocked with 5% BSA in PBS for 1 h. Cells were stained overnight at 4 °C with antibodies to BrdU (#347580, BD Biosciences) and γH2AX (# 2577, Cell Signalling Technology) and bound antibodies were detected with anti-mouse antibody Alexa Fluor 488 (#A11029, Invitrogen) and anti-rabbit antibody Alexa Fluor 594 (#A11037, Invitrogen). After mounting with antifade mounting medium containing DAPI (Vector Laboratories), slides were imaged on a ZEISS LSM 710 confocal microscope (Carl Zeiss Microscopy).

*dNTP assay* utilised a solid-phase polymerase assay modified from [44], using tritium (^3^H)-labelled substrates with commercially available dNTPs as standards, as detailed in Supplementary Methods and Supplementary Table S10.

*3D spheroid culture* was performed as [14]. After completing drug treatments, spheroids were used for western blot, dNTP assay or viability assay (CellTiter Glo 3D, Promega). For western blots, ≥3 spheroids for each condition were lysed in 3× Laemmli sample buffer (150 mM Tris-HCl pH 6.8, 0.3 mg/mL Bromophenol blue, 30% Glycerol, 9% SDS, 15% β-mercaptoethanol). For dNTP measurement, twelve spheroids per condition were extracted in 1 mL cold (−20 °C) 60% methanol, vigorously vortexed or sonicated at 4 °C (Bioruptor sonicator, Diagenode), incubated at −80 °C overnight and assayed as described in Supplementary Methods. Samples were normalised using CellTiter Glo 3D or BCA protein assay data to adjust volumes of each extract used for dNTP assay.

### In vivo experiments

Animal procedures were conducted under PPL 30/3395 and PIL IC38C8060 issued by the UK Home Office. Before Home Office submission, the Project Licence was approved by the Oxford University Animal Welfare and Ethical Review Board. As described in [14], SK-CO-1 cells (8 × 10^6^ cells/mouse) were grown as xenografts in 5–6-week-old female CD-1 immunodeficient mice (Charles River Laboratories). When tumours attained ~80–100 mm^3^, mice were randomly grouped into four groups (*N* = 5) for twice weekly intraperitoneal injection: group (1) PBS with 20% (2-Hydroxypropyl)-beta-cyclodextrin (Sigma-Aldrich), (2) PBS with 50 mg/kg MK-8776 diluted in 20% (2-Hydroxypropyl)-beta-cyclodextrin, (3) 100 mg/kg xentuzumab with 20% (2-Hydroxypropyl)-beta-cyclodextrin and (4) 100 mg/kg xentuzumab with 50 mg/kg MK-8776 in 20% (2-Hydroxypropyl)-beta-cyclodextrin. Mice were monitored regularly, tumours were measured every 2–3 days by Biomedical Services staff who were blinded to treatment allocation, and tumour volumes calculated as Volume (mm^3^) = π/6 × Length (mm) × width (mm) × height (mm).

### Statistics

Data were presented as mean ± standard error of mean (SEM), *n* = 3 independent experiments unless stated otherwise. In Graphpad Prism 8 we used two-tailed *t* test to compare two groups, one-way analysis of variance (ANOVA) for >2 groups and two-way ANOVA for proliferation, cell death and clonogenic assay dose–response curves, with post-hoc analysis and correction for multiple comparisons to assess significance at each drug concentration. *P* values <0.05 were considered statistically significant. Combination indices were calculated using CalcuSyn Software [52] and Bliss Independence [53].

## Supplementary information

XWu_Supplemental file
