# Supplementary material for: CHK1 inhibition exacerbates replication stress induced by IGF blockade
Source: Oncogene. 2021 Nov 12;41(4):476–88. doi: 10.1038/s41388-021-02080-1 (PMC8782724; doi:10.1038/s41388-021-02080-1)
Supplement: Supplementary file 1 — XWu_Supplemental file [file 41388_2021_2080_MOESM1_ESM.pdf]

WU ET AL: SUPPLEMENTAL FILE

SUPPLEMENTARY METHODS

Compound screen

The screen was performed in duplicate in 5 oestrogen receptor (ER) positive breast cancer cell lines: MCF7, ZR-75-1, KPL1, HCC1143 and T47D. Cells were seeded in 96-well plates and treated with the 60-compound custom library (Table S1) at 3 concentrations (0.1 µM, 1 µM, 10 µM) in combination with PBS (control) or 1 µM xentuzumab for 5 days using Janus liquid handling workstations (Perkin Elmer). Cell viability was determined by adding 100 µL phenol red-free DMEM (#21063029, Gibco) containing 10 µg/mL resazurin, measured on an Envison multilabel plate reader (Perkin Elmer). Data were collected and further analysed using Microsoft Excel and GraphPad Prism 8. To assess the quality of the compound screen, Z-factors were calculated using Zhang and colleagues' method (1).

$$Z = 1 - \frac{3(\sigma_P + \sigma_N)}{|\mu_P - \mu_N|}$$

Here, μ_P is the mean value of the positive control (PLK inhibitor BI-2536), μ_N is the mean value of the negative control (DMSO solvent), σ_P is the standard deviation (SD) of the positive control and σ_N is the SD of negative control.

To determine the efficacy of each compound as a single treatment or in combination with xentuzumab, the fluorescence intensities measured in duplicate plates were averaged and relative viability (compared with DMSO solvent) was calculated:

$$Relative\ viability = \frac{Fluorescence\ intensity\ of\ test\ compound\ well\ (+/-xentuzumab)}{Fluorescence\ intensity\ of\ DMSO\ well\ (+/-xentuzumab)}$$

Relative viability in DMSO control-treated well was corrected to 1. To compare the efficacies of each compound alone and in combination with xentuzumab, the following formula was used:

$$E_{compound} = \log_{10} \frac{relative\ viability\ in\ single\ treatment\ plate}{relative\ viability\ in\ combination\ treatment\ plate}$$

$E_{compound}$ = 0 indicates no combination effect ie relative viability of cells treated with the compound alone is the same as in the combination treatment plate (E= $Log_{10}(1) = 0$). $E_R > 0$ indicates increased efficacy when the compound is combined with xentuzumab (relative viability of the cells in single treatment is larger than in combination treatment), suggesting a combination effect. $E_R < 0$ indicates reduced efficacy when the compound is combined with xentuzumab (relative viability of cells treated with compound alone is less than in combination treatment). For each cell line, $E_{compound}$ values of all compounds at 0.1 µM, 1 µM, 10 µM were ranked. Z-score was calculated using a robust method based on median and median absolute deviation (MAD) as follows (2):

$$Z_{compound} = \frac{E_{compound} - Median\ (all\ \ compounds)}{MAD}$$

$$MAD = 1.4826\ \times Median\ (|E_{compound} - Median\ (all\ \ compounds)|)$$

To identify hits showing combination effects a Z-score cut-off of >2 was used, as recommended (3). Positive hits were imported into (http://bioinformatics.psb.ugent.be/webtools/Venn/) to identify overlapping hits in the 5 cell lines.

dNTP assay

The method for dNTP measurement was a solid-phase polymerase assay using tritium (^{3}H)-labelled substrates, modified from (4, 5). At least 10^6 cells were used for each condition in each

experiment. Cells were gently washed with 1 x PBS and pelleted. To extract dNTPs, 1 mL cold (-20 °C) 60% methanol was added while vortexing, and then incubated for 1 hour (or overnight) at – 80 °C. The samples were boiled at 95 °C for 3 minutes. After cooling to room temperature for ~30 minutes, samples were centrifuged at 15,000 g for 10 minutes. The supernatants were collected and dried in a speed vacuum concentrator. The solid dNTP extracts were assayed immediately or stored at – 80 °C until analysis. The polymerase reactions were performed in a streptavidin-coated 96-well plate (#15125, Thermo Scientific). The streptavidin-coated 96-well plates were first prepared by affinity capture of biotinylated oligonucleotides as templates for dNTP incorporation. The 4 oligonucleotides and the common primer for 4 dNTP incorporation reactions were designed and modified based on Sherman and Fyfe's protocol (5) and are shown in Supplementary Table S10A. The biotinylated oligonucleotides were added into the streptavidin-coated plate at the final concentration of 0.25 µM in 50 µL PBS-0.1 % Tween-20 (PBST). The plate was incubated while gently shaking at room temperature for 2 hours, allowing for biotin-streptavidin binding. After washing 3 times with 50 µL TENT buffer (40 mM Tris-HCl, 1mM EDTA, 50mM NaCl, 0.1% TWEEN-20, pH 8.0), the primer was added into the wells at 0.25 µM in 50 µL PBST. The plate was heated to 95 °C for 4 minutes and cooled to room temperature over 1 hour, allowing primer annealing to the oligonucleotide template. Commercial dNTPs (#N0446S, New England Biolabs), dATP, dTTP, dCTP and dGTP were used as assay standards and were individually diluted to final concentration 10 - 100 nM, with a sterile water blank.

To prepare the samples, solid dNTP extracts were diluted in sterile nuclease-free water, typically 500 µL for extracts from 10^6 MCF7 cells. The polymerase reaction mix components are shown in Table S3. For dATP measurement, [^{3}H]-dTTP (#NET520A250UC, PerkinElmer) was used. For dTTP, dCTP and dGTP measurement, [^{3}H]-dATP (#NET268250UC, PerkinElmer) was used. The reaction mixtures were first prepared by mixing reaction components without samples or standards and then aliquoted (37.5 µL/well) into oligonucleotide template-coated 96-well plates. The samples were added (12.5 µL/well) in triplicate wells, with blank wells containing 12.5 µL nuclease-free water (Supplementary Table S10B). Plates were incubated at room temperature for 1 hour, allowing dNTP incorporation. The contents of the wells were discarded, and wells were washed 4 times with 50 µL TENT buffer. All the TENT buffer was carefully removed after the final wash. To release the newly synthesised labelled DNA strand, 60 µL of 50 mM NaOH was added into the wells and incubated for 3 minutes at room temperature. The eluted DNA was transferred to scintillation vials containing 3 mL scintillation cocktail (OptiPhase supermix cocktail, #1200-439, PerkinElmer). Radioactivity was determined on a Beckman LSC LS6500 scintillation counter (PerkinElmer) at 1 minute counting per sample. Counts-per-minute (CPM) data were imported into Microsoft Excel for further analysis. Results were normalised by subtracting the mean blank well value then expressed as % controls.

SUPPLEMENTARY REFERENCES

1. Zhang JH, Chung TD, Oldenburg KR. A Simple Statistical Parameter for Use in Evaluation and Validation of High Throughput Screening Assays. J Biomol Screen. 1999;4(2):67-73.
2. Brideau C, Gunter B, Pikounis B, Liaw A. Improved statistical methods for hit selection in high-throughput screening. J Biomol Screen. 2003;8(6):634-47.
3. Goktug AN, Chai SC, Chen T. Data analysis approaches in high throughput screening. Drug Discovery. 2013:201-26.
4. Landoni JC, Wang L, Suomalainen A. Quantitative solid-phase assay to measure deoxynucleoside triphosphate pools. Biology Methods and Protocols. 2018;3(1):bpy011.
5. Sherman PA, Fyfe JA. Enzymatic assay for deoxyribonucleoside triphosphates using synthetic oligonucleotides as template primers. Anal Biochem. 1989;180(2):222-6.

Supplementary Table S1 Compound library.

	Compound name	Main target	Catalogue number	Supplier
**1**	ABT-888 (Veliparib)	PARP1/2	S1004	Selleck Chemicals
**2**	Deforolimus (MK-8669)	mTOR	S1022	Selleck Chemicals
**3**	Lapatinib Ditosylate	EGFR | HER2	S1028	Selleck Chemicals
**4**	VX-680 (Tozasertib)	Aurora kinase (pan A,B,C)	S1048	Selleck Chemicals
**5**	BMS-599626 (AC480)	EGFR | HER2	S1056	Selleck Chemicals
**6**	Obatoclax Mesylate	BCL-2	S1057	Selleck Chemicals
**7**	AZD2281(Olaparib)	PARP	S1060	Selleck Chemicals
**8**	Nutlin 3	Mdm2/p53	S1061	Selleck Chemicals
**9**	MK-2206	AKT	S1078	Selleck Chemicals
**10**	KU-55933	ATM	S1092	Selleck Chemicals
**11**	AG-014699 (Rucaparib)	PARP1	S1098	Selleck Chemicals
**12**	BI 2536	PLK1	S1109	Selleck Chemicals
**13**	PD0332991 (Palbociclib)	CDK4/6	S1116	Selleck Chemicals
**14**	SNS-032(BMS-387032)	CDK2, CDK7/9	S1145	Selleck Chemicals
**15**	Gemcitabine Hydrochloride	DNA synthesis	S1149	Selleck Chemicals
**16**	Roscovitine (CYC202)	CDK1/2, CDK5	S1153	Selleck Chemicals
**17**	JNJ 26854165 (Serdemetan)	p53	S1172	Selleck Chemicals
**18**	Fulvestrant	Estrogen receptor	S1191	Selleck Chemicals
**19**	CUDC-101	HDAC | EGFR | HER2	S1194	Selleck Chemicals
**20**	Irinotecan	Topoisomerase I	S1198	Selleck Chemicals
**21**	PIK-75 Hydrochloride	PI3K | DNA-PK	S1205	Selleck Chemicals
**22**	Adriamycin (Doxorubicin)	Topoisomerase II	S1208	Selleck Chemicals
**23**	Clofarabine	Ribonucleotide reductase	S1218	Selleck Chemicals
**24**	Etoposide	Topoisomerase II	S1225	Selleck Chemicals
**25**	Flavopiridol (Alvocidib)	CDK1/2, CDK4/6, CDK9	S1230	Selleck Chemicals
**26**	Camptothecin	Topoisomerase I	S1288	Selleck Chemicals
**27**	ON-01910 (Rigosertib)	PLK1	S1362	Selleck Chemicals
**28**	Ispinesib (SB-715992)	Kinesin spindle protein	S1452	Selleck Chemicals
**29**	MC1568	HDAC	S1484	Selleck Chemicals
**30**	DMSO	Solvent control	D2650	Sigma-Aldrich
**31**	AT7519	CDK1/2, CDK4/6, CDK9	S1524	Selleck Chemicals
**32**	Hesperadin	Aurora kinase B	S1529	Selleck Chemicals
**33**	BIX 02189	MEK	S1531	Selleck Chemicals
**34**	AZD7762	CHK1/2	S1532	Selleck Chemicals
**35**	PD318088	MEK	S1568	Selleck Chemicals
**36**	KU-60019	ATM	S1570	Selleck Chemicals
**37**	BS-181 hydrochloride	CDK7	S1572	Selleck Chemicals
**38**	LY500307 (Erteberel)	Estrogen receptor β	S1598	Selleck Chemicals
**39**	Cytarabine (Cytosar-U)	DNA synthesis	S1648	Selleck Chemicals
**40**	Flucytosine (Ancobon)	DNA/RNA synthesis	S1666	Selleck Chemicals
**41**	Tamoxifen Citrate	Estrogen receptor	S1972	Selleck Chemicals
**42**	MLN2238 (Ixazomib)	Proteasome inhibitor	S2180	Selleck Chemicals
**43**	SB 743921	Kinesin spindle protein	S2182	Selleck Chemicals
**44**	Indirubin	CDK pan| GSK-3β	S2386	Selleck Chemicals
**45**	RITA (NSC 652287)	p53 inhibitor	S2781	Selleck Chemicals
**46**	BMN673 (Talazoparib)	PARP1/2	S7048	Selleck Chemicals
**47**	CO-1686 (Rociletinib)	EGFR	S7284	Selleck Chemicals
**48**	E3330	APE1	S7445	Selleck Chemicals
**49**	CRT0044876	APE1	S7449	Selleck Chemicals
**50**	VE-821	ATR	S8007	Selleck Chemicals
**51**	B02	RAD51	SML0364	Sigma-Aldrich
**52**	Mirin	MRN complex	S8096	Selleck Chemicals
**53**	Ro-3306	CDK1	15149	Cayman Chemical
**54**	SCH-900776 (MK-8776)	CHK1	S2735	Selleck Chemicals
**55**	MK-1775 (Adavosertib)	WEE1	1494	Axon Medchem
**56**	Abemaciclib (LY2835219)	CDK4/6	S7158	Selleck Chemicals
**57**	ML216	BLM	SML0661	Sigma-Aldrich
**58**	AZD5363 (Capivasertib)	AKT	S8019	Selleck Chemicals
**59**	Celastrol	NF-κB	3203	Tocris Bioscience
**60**	NSC 19630	WRN Helicase	681647	Calbiochem

Supplementary Table S2. Top ranked compounds in MCF7

Rank in screen	Compound[target]	Z-score
1	MK-2206[AKT]	2.99
2	Lapatinib[EGFR, HER2]	2.79
3	CUDC-101[EGFR,HER2]	2.67
4	KU-55933[ATM]	2.50
5	Ro-3306[CDK1]	2.35
6	PD-318088[MEK]	2.30
7	BMS-599626[EGFR, HER2]	2.17
8	B02[RAD51]	2.04

Supplementary Table S3. Top ranked compounds in ZR-75-1

Rank in screen	Compound[target]	Z-score
1	Lapatinib[EGFR, HER2]	4.79
2	Mirin[MRN]	3.41
3	Celastrol[HSP90]	3.01
4	AG-014699[PARP]	2.99
5	KU-55933[ATM]	2.99
6	KU-60019[ATM]	2.46
7	B02[RAD51]	2.40
8	BMS-599626[EGFR, HER2]	2.37
9	BIX-02189[MEK]	2.31
10	Deforolimus[mTOR]	2.19
11	Tamoxifen Citrate[ER]	2.18
12	ABT-888[PARP]	2.09

Supplementary Table S4. Top ranked compounds in T47D

Rank in screen	Compound[target]	Z-score
1	MK-8776[CHK1]	3.38
2	CUDC-101[EGFR,HER2]	3.00
3	Lapatinib[EGFR, HER2]	2.97
4	BMS-599626[EGFR, HER2]	2.95
5	PD0332991[CDK4/6]	2.26
6	AG-014699[PARP]	2.17
7	AZD-7762[CHK]	2.16
8	Deforolimus[mTOR]	2.09

Supplementary Table S5. Top ranked compounds in KPL1

Rank in screen	Compound[target]	Z-score
1	Obatoclax Mesylate[BCL2]	4.46
2	Fulvestrant[ER]	3.94
3	Lapatinib[EGFR, HER2]	3.73
4	JNJ 26854165[p53]	3.69
5	B02[RAD51]	3.33
6	BMS-599626[EGFR, HER2]	3.28
7	AG-014699[PARP]	2.54
8	MK-1775[WEE1]	2.42
9	MK-8776[CHK1]	2.42
10	LY500307[Erβ]	2.24
11	Nutlin 3[MDM2/P53]	2.03

Supplementary Table S6. Top ranked compounds in HCC1143

Rank in screen	Compound[target]	Z-score
1	BMS-599626[EGFR, HER2]	3.35
2	PIK-75[PI3K, DNA-PK]	2.63
3	MK-2206[AKT]	2.54
4	Fulvestrant[ER]	2.52
5	Mirin[MRN]	2.49
6	Nutlin 3[MDM2/P53]	2.43
7	Lapatinib[EGFR, HER2]	2.39
8	MK-8776[CHK1]	2.37
9	Obatoclax Mesylate[BCL2]	2.33
10	KU-55933[ATM]	2.30
11	ABT-888[PARP]	2.22
12	Olaparib[PARP]	2.21
13	AG-014699[PARP]	2.00

Supplementary Table S7: Screen Z-scores.

(A) Compounds in combination with xentuzumab

	MK-8776[CHK1]			VE-821[ATR]		
Concentration	0.1µM	1µM	10µM	0.1µM	1µM	10µM
MCF7	-0.52558863	-0.58070355	-0.31127958	-0.18795124	0.31898495	-0.66801267
ZR-75-1	-1.12583091	-0.76600392	1.6080418	-0.07841656	0.4730385	0.48435506
T47D	-1.28208934	-0.97281174	3.37557803	-0.70137326	-0.3333903	0.06142209
KPL1	-0.9668267	0.38021108	2.41788225	-0.49798153	0.06506156	1.8020468
HCC1143	-0.76625243	-2.43587902	2.37295534	0.21545005	0.32560734	0.98250228

	KU-55933[ATM]			KU-60019[ATM]		
Concentration	0.1µM	1µM	10µM	0.1µM	1µM	10µM
MCF7	0.89361619	1.47916954	2.4967716	0.02401269	1.21669392	0.64224773
ZR-75-1	0.56524968	1.81504501	2.98540889	-0.21380563	1.37279592	2.46459423
T47D	-0.41542171	0.34807846	0.92793973	-0.58508496	-0.16266135	1.14635982
KPL1	-0.03243799	0.25065267	1.98506005	-0.99185084	-0.22904266	1.86189861
HCC1143	0.55773484	1.71856928	2.3012674	-0.70824174	-0.00758993	1.99681868

	ABT-888[PARP]			AG-014699[PARP]			Olaparib[PARP]		
Concentration	0.1µM	1µM	10µM	0.1µM	1µM	10µM	0.1µM	1µM	10µM
MCF7	0.32627884	0.54400835	0.86285447	0.26222563	-0.23106453	0.30264511	0.41128808	1.038249	-0.5328665
ZR-75-1	-1.0857309	0.06345538	2.08646216	-0.34871653	0.49864385	2.99267597	0.21063932	0.50315178	0.94888183
T47D	-0.76724874	0.01325059	1.55793302	-0.44844668	0.00858582	2.1662672	0.03643708	0.17535293	1.24647015
KPL1	-1.11114177	-0.03583971	1.17507158	-0.99660079	-0.14678122	2.54266241	-1.02343601	-0.2454942	1.51892955
HCC1143	-0.07600227	0.66134152	2.22187448	-0.04166981	0.22082605	2.00183434	0.40770549	1.0185815	2.21102338

(B) Compounds alone

	MK-8776[CHK1]			VE-821[ATR]		
Concentration	0.1µM	1µM	10µM	0.1µM	1µM	10µM
MCF7	0.1027196	-0.45897388	-0.65239934	0.67881867	0.68821723	-0.4453635
ZR-75-1	0.79043217	0.09869132	-0.28664659	0.80197118	1.22193454	0.71802523
T47D	0.97019181	0.98257457	-0.03603378	1.08178948	1.45699792	0.25927729
KPL1	-0.38663822	-1.41687531	2.57893178	0.27577948	-0.18559874	-0.10164495
HCC1143	0.66396749	-0.61950223	-0.53973555	1.04751572	1.38883039	0.45919907

	KU-55933[ATM]			KU-60019[ATM]		
Concentration	0.1µM	1µM	10µM	0.1µM	1µM	10µM
MCF7	1.12561137	1.30724499	1.41231151	0.49026196	0.94871051	-0.41813918
ZR-75-1	0.81388859	1.00985285	0.28897645	0.97495164	1.09220686	0.13598062
T47D	0.76958375	1.22796231	0.81048178	0.662952	0.77318758	0.21952137
KPL1	2.03317385	2.49184906	0.96711243	0.00911362	0.46742972	0.55508268
HCC1143	1.01825939	1.36827939	1.2176773	0.45327485	0.7859577	0.7561338

	ABT-888[PARP]			AG-014699[PARP]			Olaparib[PARP]		
Concentration	0.1µM	1µM	10µM	0.1µM	1µM	10µM	0.1µM	1µM	10µM
MCF7	0.87353656	0.97025621	1.86075002	0.65504014	0.52807472	0.1968379	0.65687847	0.69571879	-0.0671209
ZR-75-1	0.67970028	1.16491621	1.92881036	0.42464809	0.56021659	0.59306477	0.70519464	0.82947693	0.54231807
T47D	0.64088792	1.03889152	1.71875547	0.53455236	0.95540143	0.81853907	0.5731669	0.77102781	0.89742876
KPL1	1.16550028	1.58768003	0.42956572	0.60881294	0.93214741	1.27870742	0.55704995	0.37945266	-0.1699676
HCC1143	0.82808009	1.03579648	1.73422956	0.5843704	0.71431175	0.44554518	0.68055784	0.92129549	0.99289959

(A) Z-scores for selected compounds in combination with xentuzumab. Screen hits identified by Z-score >2 (marked in red) in combination with xentuzumab. **(B)** Z-scores for compounds alone.

Supplementary Table S8. Synergy tested by Bliss Independence

		MK-8776 (µM)			
		0.3	1	3	10
Xentuzumab (nM)	100	0.73 ± 0.09	0.69 ± 0.09	0.74 ± 0.07	0.92 ± 0.02
	300	0.65 ± 0.06	0.42 ± 0.13	0.54 ± 0.09	0.75 ± 0.04
	1000	0.39 ± 0.07	0.49 ± 0.10	0.60 ± 0.08	0.72 ± 0.02

Using 3D CellTiter Glo data as used for synergy testing by the Chou and Talalay method (Figure 6D), we also tested synergy by Bliss Independence. First, we calculated the fraction affected (1-viable fraction), then used the following equation: Ei = (EA +EB) - (EA x EB), where Ei is the predicted effect (fraction affected) by the combination of drugs A and B if they act additively and independently, and EA and EB are the observed fraction affected by each drug alone. The results are expressed as mean ± SEM fraction of the observed effect of the combination (ie predicted/observed), from three independent experiments each with 3 technical replicates. Values <0.8 indicate synergy.

Supplementary Table S9. Antibodies used for western blot and immunofluorescence

Antibody	Species	Dilution	Catalogue #	Supplier
IGF-1R β	Rabbit	1:1000	3027	Cell Signaling Technology
pIGF-1Rβ(Tyr1135/1136) /pINSR β (Tyr1150/1151)	Rabbit	1:500	3024	Cell Signaling Technology
AKT	Rabbit	1:1000	9272	Cell Signaling Technology
pATK (S473)	Rabbit	1:1000	4060	Cell Signaling Technology
ERK	Mouse	1:1000	4696	Cell Signaling Technology
pERK (T202/Y204)	Rabbit	1:1000	9101	Cell Signaling Technology
RRM2	Goat	1:2000	SC-10844	Santa Cruz Biotechnology
CHK1	Mouse	1:1000	SC-8408	Santa Cruz Biotechnology
pCHK1 (S296)	Rabbit	1:1000	90178	Cell Signaling Technology
pCHK1 (S345)	Rabbit	1:1000	2348	Cell Signaling Technology
CDK1	Rabbit	1:1000	77055	Cell Signaling Technology
pCDK1 (Y15)	Rabbit	1:1000	4539	Cell Signaling Technology
β-tubulin	Mouse	1:3000	86298	Cell Signaling Technology
β-actin	Mouse	1:3000	A1978	Sigma-Aldrich
γH2AX (S139)	Rabbit	1:500	2577	Cell Signaling Technology
BrdU	Mouse	1:500	347580	BD Biosciences

Supplementary Table S10. Components of dNTP assay.

(A) Oligonucleotides

	Sequence (5' – 3')	Length
Primer	CCCGCCTCCACCGCC	21 bp
Oligo for dATP	[B]AAATAAATAAATAAATAAATGGCGGTGGAGGCGGG	41 bp
Oligo for dTTP	[B]TTATTATTATTATTATTAGGCGGTGGAGGCGGG	39 bp
Oligo for dCTP	[B]TTTGTTTGTTTGTTTGTTTGGGCGGTGGAGGCGGG	41 bp
Oligo for dGTP	[B]TTTCTTTCTTTCTTTCTTTCGGCGGTGGAGGCGGG	41 bp

(B) Reaction components

	Volume (µL)	Final concentration
10x polymerase buffer	5	1x
Klenow polymerase (10 U/ µL)	0.125	1.25 U
DTT (500 mM)	0.5	5 mM
[^{3}H]-dATP/[^{3}H]-dTTP	2	[^{3}H]-dATP: 0.04 uCi/ µL
		[^{3}H]-dTTP: 0.1 uCi/ µL
Sample/standard	12.5	
Sterile water	29.875	
Total	50	

(A) The 5'-biotinylated (B) oligonucleotides were synthesised and HPLC-purified at Integrated DNA Technologies (IDT), diluted to 5 µM in sterile H_2O, aliquoted and stored at –20°C. **(B)** DNA polymerase reaction components for dNTP assay.

Supplementary Figures

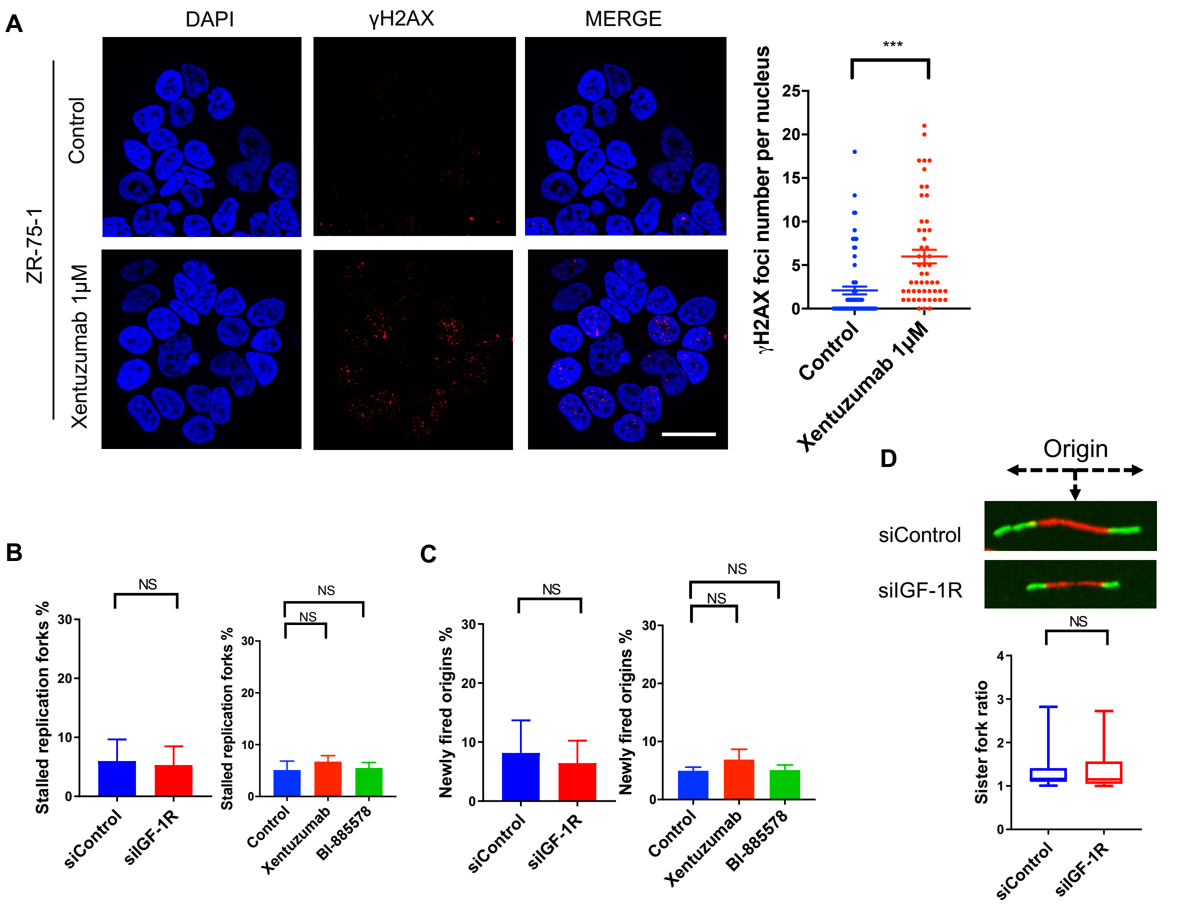

Supplementary Figure S1. IGF blockade induces tolerable replication stress. (A) Representative images of γH2AX immunostaining in ZR-75-1 cells treated with 1 μM xentuzumab for 72 hours. Scale bar: 20 μm. Quantification of γH2AX foci per nucleus (>50 cells) shown on the right. Data represent mean ± SEM, pooled from 2 independent experiments. **(B)** Quantification of stalled replication forks (CldU/red-only tracts) as percentages of the total number of tracts (as imaged in Figure 1D-E). **(C)** Quantification of newly fired origins (IdU/green-only tracts) as percentages of the total number of tracts (from Figure 1D-E). **(D)** Representative images of sister forks from MCF7 cells 48 hours after siRNA transfection. Quantification of ratios of sister forks (> 20 pairs of sister forks in Figure 1D-E) shown below as median with 25th and 75th percentiles; whiskers mark smallest and largest values.

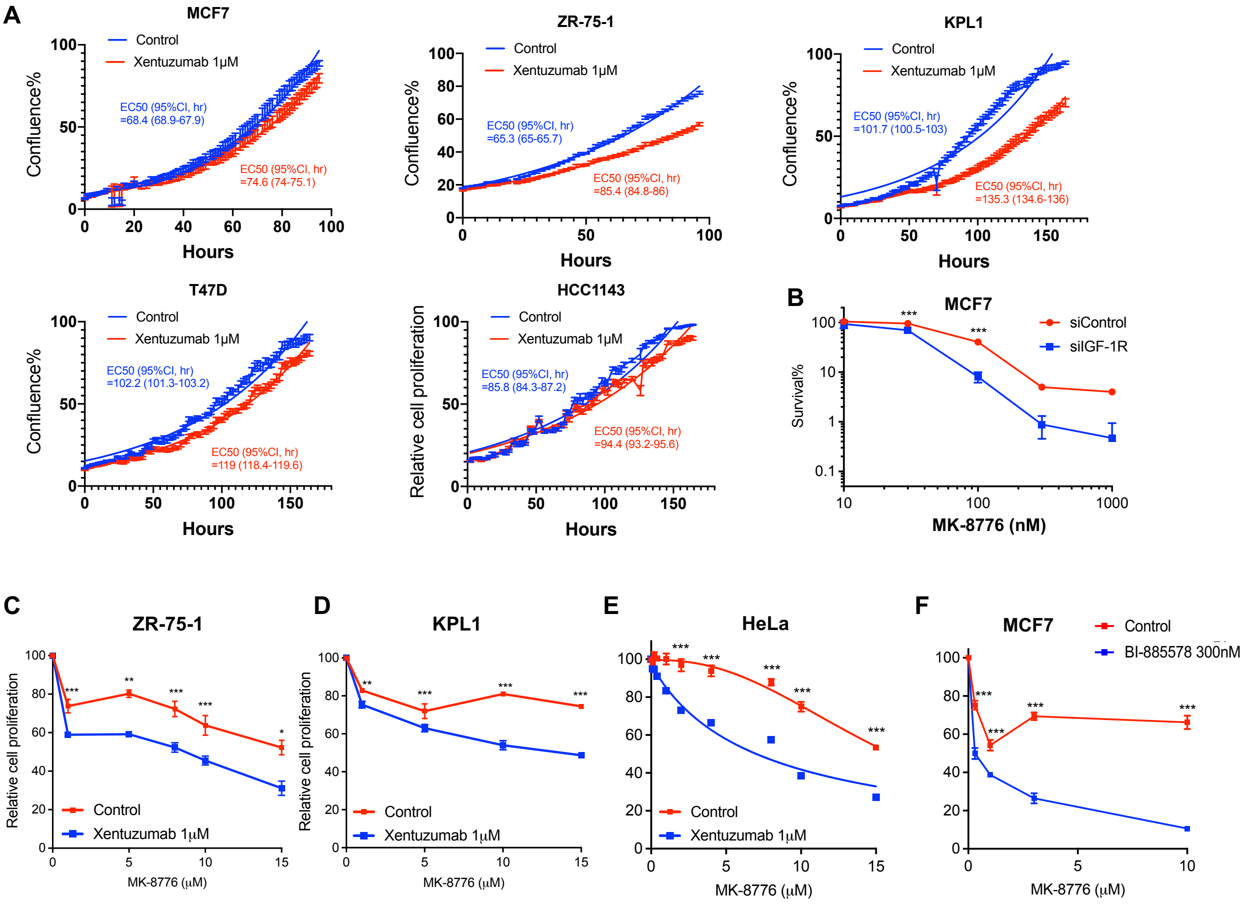

Supplementary Figure S2. Incucyte analysis of xentuzumab treatment and validation of CHK1 as screen hit. (A) MCF7, ZR-75-1, KPL1, T47D, HCC1143 cells were exposed to 1 μM xentuzumab for 4-6 days and growth curves were obtained using the IncuCyte live cell analysis imaging system. Half maximal effective concentrations (EC_{50}) and 95% confidence intervals were determined in Graphpad Prism 8. **(B)** MCF7 cells were transfected with siControl or siIGF-1R, after 24 hours were reseeded for clonogenic assay and then exposed to solvent (control) or MK-8776 for 7 days. Cell survival expressed as % viability of solvent treated siRNA-transfected cells. Data represent mean ± SEM, pooled from 3 independent experiments. **(C-F)** ZR-75-1 (C), KPL1 (D), and HeLa cells (E) were exposed to xentuzumab and MK-8776 for 5 days, and MCF7 cells (F) were exposed to BI-885578 and MK-8776 for 5 days, followed by cell viability assay. Data represent mean ± SEM % viability of solvent-treated controls. Data in B-F were analysed by 2-way ANOVA, showing that targeting the IGF axis with IGF-1R depletion (B), xentuzumab (C-E) or BI-885578 (F) caused a significant shift in the MK-8776 dose-response curve ($P<0.001$ in each case).

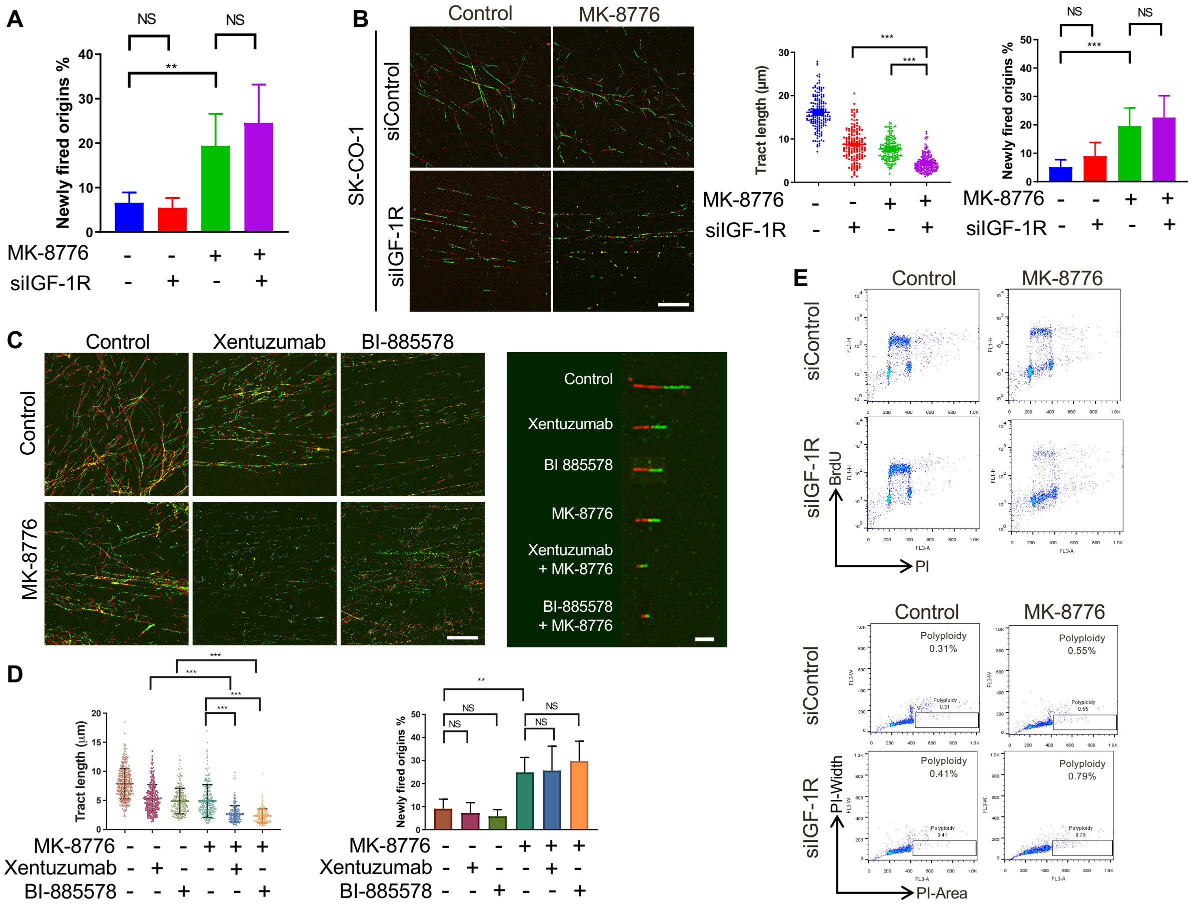

Supplementary Figure S3. CHK1 inhibition induces replication catastrophe in IGF-1R depleted or inhibited cells. (A) Quantification of newly fired origins (IdU (green)-only tracts) as percentages of total number of tracts (from Figure 3A). **(B)** Representative images of DNA fiber tracts: (CldU, red; IdU, green) in SK-CO-1 cells transfected with siControl or siIGF-1R for 24 hours and exposed to solvent or 300 nM MK-8776 for 24 hours. Scale bar: 20 µm. Graphs to right: quantification of fiber tract length (>150 tracts) and newly fired origins (IdU/green-only tracts as percentages of the total number of tracts (≥ 5 images). **(C)** Images of DNA fiber tracts in MCF7 cells exposed to 1 µM xentuzumab or 300 nM BI-885578 in combination with solvent or 3 µM MK-8776 for 24 hours. Scale bar: 20 µm. To right: representative fibers under each treatment condition. Scale bar: 2 µm. **(D)** Quantification of fiber tract length (>150 tracts) and newly fired origins (IdU/green-only tracts) as percentages of the total number of tracts. Data (mean ± SEM) are from one of 3 independent experiments with similar results. **(E)** Flow cytometry analysis of cell cycle data from Figure 3B, ungated to quantify polyploid cells with >4N DNA content.

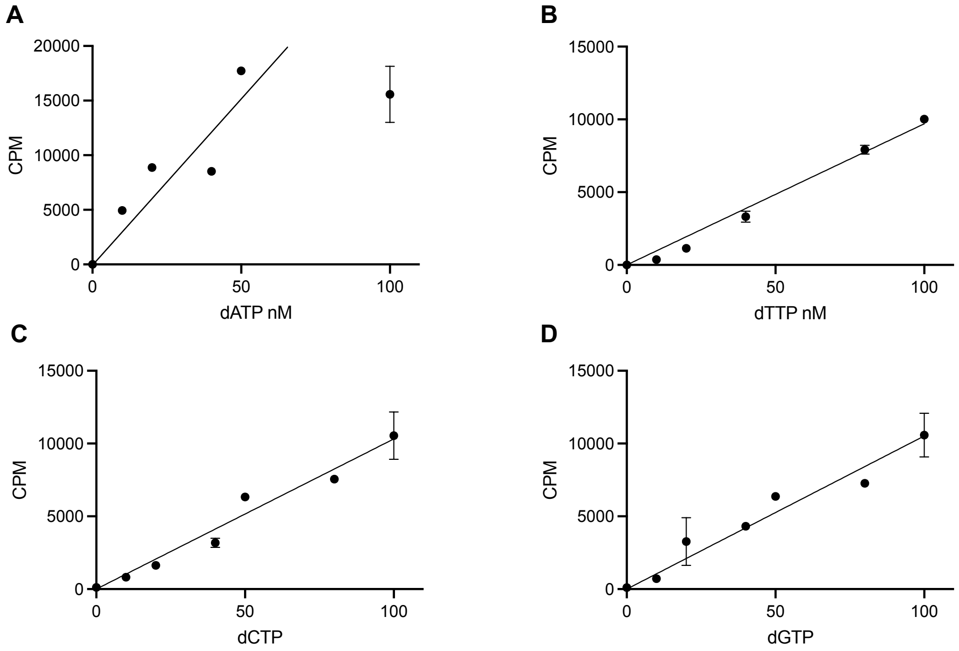

Supplementary Figure S4. Performance of dNTP standards in dNTP assay. Commercially available dATP, dTTP, dCTP and dGTP were used as assay standards as described in Supplementary Methods. Each dNTP was individually diluted to create standards: **(A)** dATP, **(B)** dTTP, **(C)** dCTP, **(D)** dGTP. Through initial tests, the optimal measurement range was determined to be 10 - 50 nM for dATP and 10 - 100 nM for dTTP, dCTP and dGTP, because radioactivity showed linear increase with dNTP concentration within this range. For data shown in Figure 4B and 6G the amounts of input extracts used for each assay were adjusted to ensure that readouts were in the linear range, and were normalised as described in Supplementary Methods.

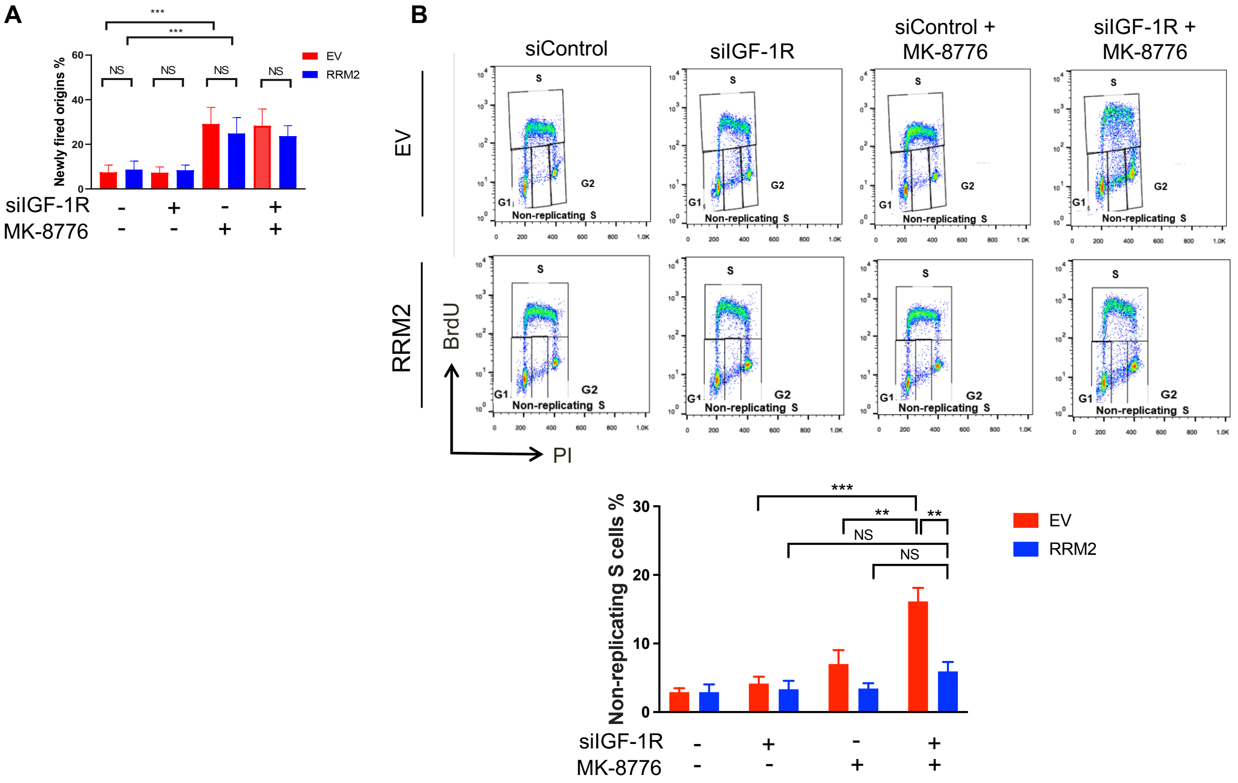

Supplementary Figure S5. RRM2 overexpression rescues replication catastrophe induced by CHK1 inhibition and IGF-1R depletion. (A) Quantification of newly fired origins (IdU/green-only tracts) as percentages of the total number of tracts (≥ 5 images from Figure 5A). Data represent mean ± SEM. **(B)** Cell cycle distribution of EV control cells and RRM2-overexpressing cells transfected with siControl or siIGF-1R for 24 hours and then exposed to solvent or 1 µM MK-8776 for 24 hours. Graph below shows quantification of non-replicating S phase cells. Data represent mean ± SD, pooled from 3 independent experiments.

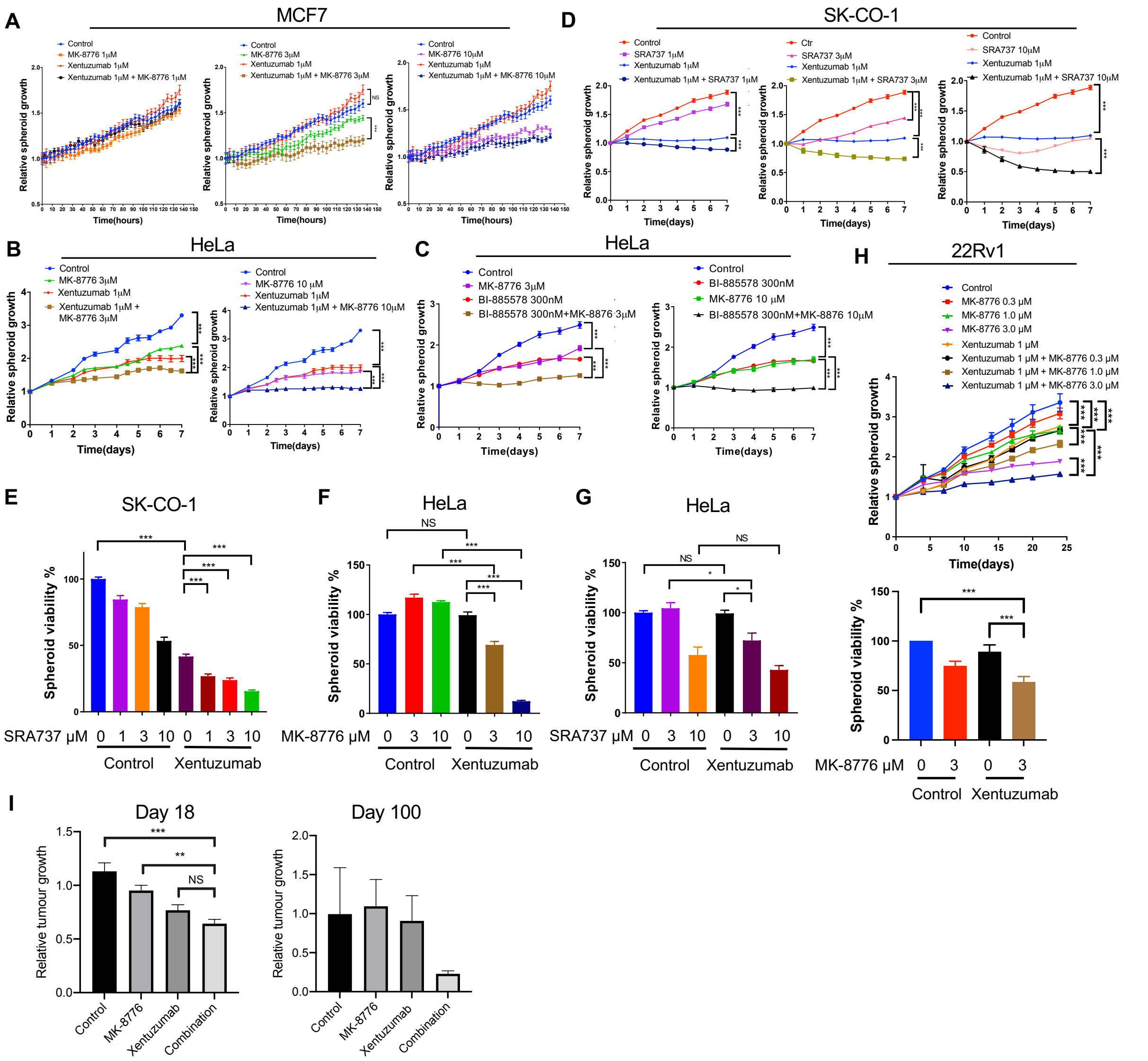

Supplementary Figure S6. IGF inhibited cells are more sensitive to CHK1 inhibition in 3D spheroid model. (A) MCF7 spheroids were treated with xentuzumab and MK-8776 for 6 days, and spheroid growth curves were analysed using spheroid size measured on IncuCyte Live Cell Analysis Imaging System. **(B, C)** HeLa spheroids were treated with MK-8776 and xentuzumab (B) or BI-885578 (C) for 7 days. Spheroid size was measured on the GelCount (Oxford Optronix) and analysed using ImageJ. **(D)** SK-CO-1 spheroids were exposed to xentuzumab and SRA737 for 7 days, spheroid size was measured as B-C. Data in A-D (mean ± SEM of 6 technical replicates) expressed as % spheroid size on Day 0 from one experiment. **(E-G)** SK-CO-1 spheroids (E) and HeLa spheroids (F, G) were treated with 1 µM xentuzumab and MK-8776 (F) or SRA737 (E, G) for 7 days, followed by CellTiter Glo 3D viability assay. Data are expressed as % solvent-treated controls and represent mean ± SEM (3 technical replicate spheroids). **(H)** 22Rv1 cells were treated with MK-8776 and xentuzumab. Upper: spheroid size (GelCount) was measured every 2-3 days, with a significant overall treatment effect and significantly reduced growth rates between xentuzumab or 1.0 or 3 µM MK-8776 alone and in combination (P<0.001 by 2-way ANOVA for each comparison). Lower: following size monitoring spheroid viability was assayed using Celltiter-Glo 3D. **(I)** Mice bearing SK-CO-1 xenografts (N=5/group) were treated twice weekly with solvent, xentuzumab, MK-8776 or the combination. After 18 days treatment, the mice were monitored for a total of 100 days. Graphs: relative tumour growth compared with Day 1 of treatment.

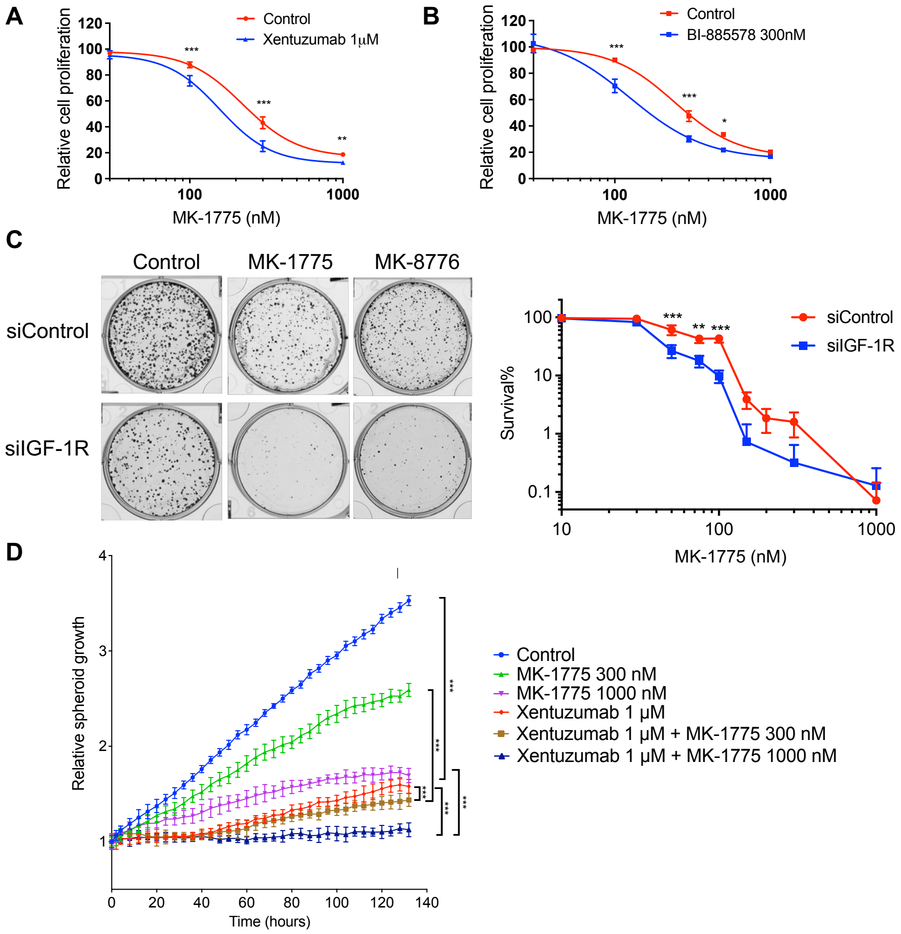

Supplementary Figure S7. Co-targeting of IGF axis and WEE1 induces combination effects in viability, clonogenic and spheroid assays. (A-B) MCF7 cells were exposed to MK-1775 in the presence or absence of 1 μM xentuzumab (A) or 300 nM BI-885578 (B) for 5 days, followed by assay of cell viability, expressed as % viability of siControl cells. Two-way ANOVA showed significant differences in the response to MK-1775 in both dose-response curves (P<0.001). **(C)** MCF7 cells were transfected with siControl or siIGF-1R, after 24 hours were reseeded for clonogenic assay and then exposed to solvent (control), MK-8776 or MK-1775 for 7 days. Representative 6-well plate showing surviving colonies following siControl or siIGF-1R transfection and treatment with solvent (control), 50 nM MK-1775 or 100 nM MK-8776. Graph to right: cell survival expressed as % viability of solvent treated siRNA-transfected cells. Data represent mean ± SEM, pooled from 3 independent experiments. By 2-way ANOVA, IGF-1R depletion caused a significant shift in the MK-1775 dose-response curve (P<0.001). **(D)** SK-CO-1 spheroids were exposed to solvent or MK-1775 in the presence or absence of 1 μM xentuzumab for 6 days. Spheroid size was measured on the IncuCyte Live Cell Analysis Imaging System and expressed as % spheroid size on Day 0, mean ± SEM from one experiment with 6 technical replicates. Two-way ANOVA confirmed a significant overall treatment effect, with significantly reduced growth rates between xentuzumab or 300 or 1000 nM MK-1775 alone and in combination (P<0.001 for each comparison).
